# Genetically engineered probiotics as catalytic glucose depriver for tumor starvation therapy

**DOI:** 10.1016/j.mtbio.2022.100515

**Published:** 2022-12-15

**Authors:** Penghao Ji, Bolin An, Zhongming Jie, Liping Wang, Shuwen Qiu, Changhao Ge, Qihui Wu, Jianlin Shi, Minfeng Huo

**Affiliations:** a Shanghai Tenth People's Hospital, Shanghai Frontiers Science Center of Nanocatalytic Medicine, School of Medicine, Tongji University, Shanghai, 200072, PR China; b State Key Laboratory of High Performance Ceramics and Superfine Microstructure, Shanghai Institute of Ceramics Chinese Academy of Sciences, Research Unit of Nanocatalytic Medicine in Specific Therapy for Serious Disease, Chinese Academy of Medical Sciences (2021RU012), Shanghai, 200050, PR China; c Center of Materials Science and Optoelectronics Engineering, University of Chinese Academy of Sciences, Beijing, 100049, PR China; d Center for Materials Synthetic Biology, Shenzhen Institute of Synthetic Biology, Shenzhen Institute of Advanced Technology, Chinese Academy of Sciences, Shenzhen, 518055, China; e CAS Key Laboratory of Quantitative Engineering Biology, Materials Synthetic Biology Center, Shenzhen Institute of Synthetic Biology, Shenzhen Institute of Advanced Technology, Chinese Academy of Sciences, Shenzhen, 518055, PR China; f School of Physical Science and Technology, Shanghai Tech University, Shanghai, 201210, PR China; g Translational Research Institute of Brain and Brain-Like Intelligence, Shanghai Fourth People's Hospital Affiliated to Tongji University School of Medicine, Shanghai, 200081, PR China

**Keywords:** Metabolic intervention, Tumor targeting, Programmable living biomaterials, Glucose deprivation, Autophagy

## Abstract

Cancer cells predominantly adapt the frequent but less efficient glycolytic process to produce ATPs rather than the highly efficient oxidative phosphorylation pathway. Such a regulated metabolic pattern in cancer cells offers promising therapeutic opportunities to kill tumors by glucose depletion or glycolysis blockade. In addition, to guarantee tumor-specific therapeutic targets, effective tumor-homing, accumulation, and retention strategies toward tumor regions should be elaborately designed. In the present work, genetically engineered tumor-targeting microbes (transgenic microorganism EcM-GDH (*Escherichia coli* MG1655) expressing exogenous glucose dehydrogenase (GDH) have been constructed to competitively deprive tumors of glucose nutrition for metabolic intervention and starvation therapy. Our results show that the engineered EcM-GDH can effectively deplete glucose and trigger pro-death autophagy and p53-initiated apoptosis in colorectal tumor cells/tissues both *in vitro* and *in vivo*. The present design illuminates the promising prospects for genetically engineered microbes in metabolic intervention therapeutics against malignant tumors based on catalytically nutrient deprivation, establishing an attractive probiotic therapeutic strategy with high effectiveness and biocompatibility.

## Graphical abstract

**Figure:**
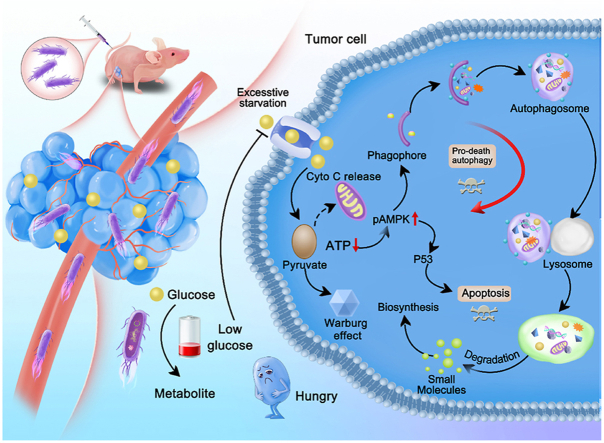

## 1 Introduction

Aerobic glycolysis is the most typical hallmark of metabolic regulation in cancer cells. Even under normoxic conditions, cancer cells primarily use the less efficient glycolytic process rather than, the more efficient oxidative phosphorylation pathway to generate energy. Such metabolic regulation of tumors results in a high level of glucose uptake to support various cellular activities, including proliferation [1]. This phenomenon, first observed by Nobel laureate Otto Warburg [2], has inspired researchers to construct effective anti-tumor strategies that target the glucose or glycolytic pathway [3]. For instance, as a glycolysis inhibitor, 2-deoxyglucose (a glucose analog) has shown promise in impeding the proliferation of cancer cells [4]. Our group previously reported that with glycolysis inhibition, 2-deoxyglucose injection combined with black phosphorus nanosheets could cooperatively induce tumor autophagy and suppress tumor progression [5]. Glucose oxidase, a glucose-specific consuming enzyme, can effectively deplete glucose for H_2_O_2_ production, benefiting the subsequent cascade reaction for Fenton therapeutics [6]. These paradigms preliminarily validate the consequence of glucose depletion for metabolic intervention against tumor cells, starving the cells to compromise with high biocompatibility. Nevertheless, effective tumor-targeting design is still limited.

Fundamental studies reveal that intravenous administrated anaerobic bacteria (such as *Escherichia*, *Shewanellaceae*, *Bifidobacterium,* and *Salmonella*) are capable of specifically targeting the tumor areas, owing to the anaerobic tropism of the bacterial cells and hypoxic and immunosuppressive tumor microenvironment [7]. A pioneering attempt to use live bacteria to treat tumors was achieved by surgeon William Coley in 1868 when he found substantial tumor suppression in sarcoma patients after infection with *Streptococcus pyogenes* [8]. Despite the surprising anti-tumor findings, this therapeutic strategy was not widely accepted at the time, mainly due to biosafety concerns. Several types of the pathogenic bacteria (e.g., *Salmonella typhimurium*) have also been shown to inhibit the growth of tumor cells. However, the innate toxicity of the bacteria may cause severe biocompatibility and biosafety considerations. Recently, commensal probiotics have received extensive research interest for their underlying therapeutic potentials and prospects in diverse gastrointestinal diseases, mental disorders, and tumors [9]. The application of these probiotics in treating tumors is one of the most promising and appealing strategies in recent advances – probiotic therapeutics. Lu and co-workers engineered the clinically approved sonosensitizer (hematoporphyrin monomethyl ether) onto the *Bifidobacteria Longum* for tumor-targeted sonodynamic immunotherapy, achieving effective tumor accumulation and xenograft destruction with satisfied biocompatibility [10]. Xie and co-workers constructed drug-conjugated bacterial carriers by attaching doxorubicin to *E. coli* Nissle 1917 using the pH labile *cis*-acetic anhydride linker. The synthetic hybrids could induce apoptosis against tumor cells through tumor-specific accumulation and tumor-environment-responsive drug release [11]. However, these cargo-loaded probiotic cells can only realize one-way delivery of the therapeutic factors to the tumor cells, and sustainable therapeutic effects from these probiotic microbes have not been demonstrated.

Synthetic biology is a frontier field at the intersection of multiple disciplines that aims to innovate biological systems by rationally rewriting the genetic information of life [12]. From the metabolic fermentation of value-added chemicals and living biotherapeutics construction to the engineering of living functional materials, synthetic biology has recently spawned an increasing number of industrial and clinical applications [13]. For disease treatment, by integrating genetic material that encodes the synthesis of therapeutic drugs into living systems (e.g., bacteria, fungi, cells), engineered living systems can perform the tailored therapeutic functions (e.g., tumor intervention) endowed by humans [14]. Many previous works have described the employment of genetically engineered bacteria or cells to treat cancers. For example, Mikhail and his colleagues developed a focused ultrasound-activatable therapeutic approach by engineering heat-responsive *E. coli* to express immune checkpoint inhibitors (αCTLA-4 and αPD-L1) for effective cancer immunotherapy [15]. Researchers from Nie's group genetically modified the bacteria *E. coli* Top10 strain to controllably express a specific tumor antigen (Fc fragment of mouse immunoglobulin G) fused with the protein cytolysin A on the surface of outer membrane vesicles. The bacteria could serve for antigen presentation, stimulating the adaptive immune responses for augmented tumor therapeutics and anti-metastasis [16]. As compared to the physical or chemical attachments of the substances, genetic engineering-based production of the therapeutic substances could not only avoid the uncontrollable detachments during *in vivo* administration, but also achieve the continuous enrichments of the therapeutic substances along with the proliferation of the probiotics. Therefore, these advances in genetic engineering and probiotics' applications are attracting growing interest due to their diverse and feasible designability. Nevertheless, few reports have focused on the genetic engineering of microbes in the applications of metabolic intervention against tumors.

**Scheme 1:**
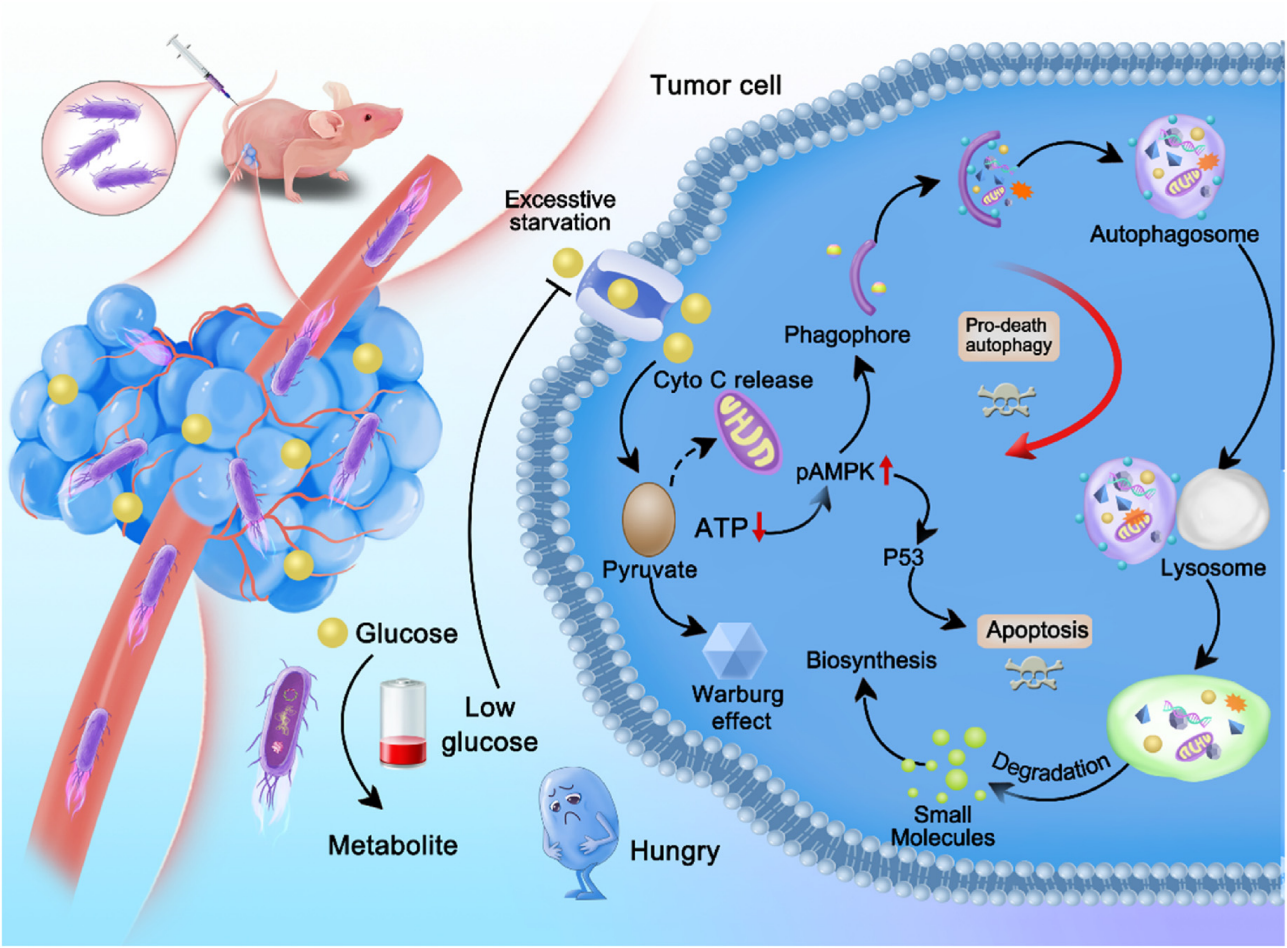
Schematic illustration for EcM-GDH to induce pro-death autophagy and apoptosis against the tumor.

In the present work, we have constructed EcM-GDH, a transgenic microorganism that continuously synthesizes glucose dehydrogenase, which can actively target tumor regions and competitively deprive glucose nutrition. The genetically programmed microbe EcM-GDH exhibits potent glucose deprivation that can block the energy supply against colorectal tumor cells/tissues, leading to dominant tumor regression with devastating effects through pro-death autophagic and p53-initiated apoptotic pathways (Scheme 1b). The paradigm of connecting catalytic medicine and synthetic biology technologies reported in this work illuminates a promising future for genetically engineered living systems in metabolic intervention therapies against malignancies, shedding light on the way to Richard Feynman's perspective envisions – “swallow the surgeon”.

## 2 Result and discussion

**Fig. 1:**
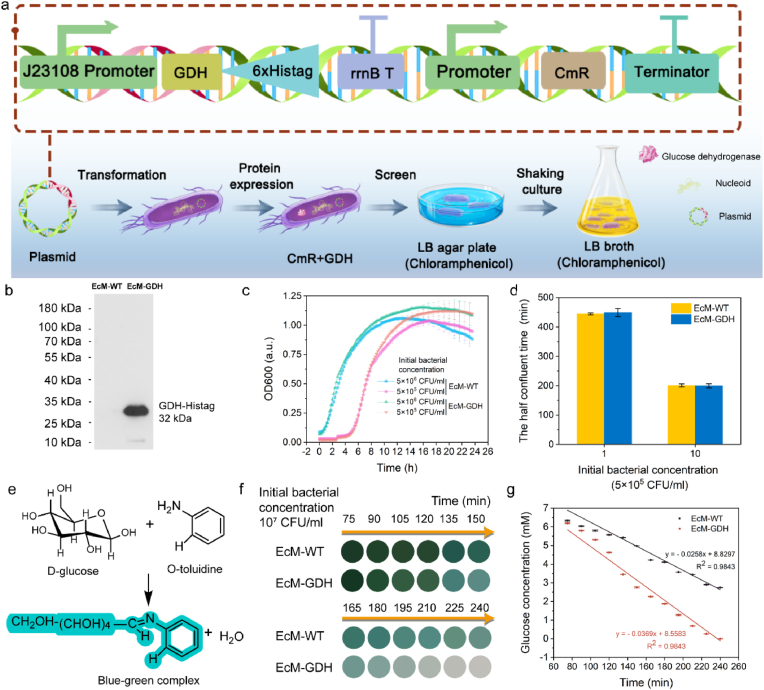
**a,** Schematic illustration of EcM-GDH construction. **b,** GDH-Histag protein expression of EcM-WT and EcM-GDH. **c,** Bacterial growth curves of EcM-WT and EcM-GDH in LB broth medium for 24 ​h. Data are expressed as means ​± ​s.d. (n ​= ​3, biological replicates). **d,** Comparison of the half confluent time for EcM-WT and EcM-GDH in LB broth medium. Data are expressed as means ​± ​s.d. (n ​= ​3, biological replicates). **e,** Schematic illustration of the chromogenic mechanism of the glucose detection kit. **f,** Images of the chromogenic assays from the mediums treated with EcM-WT or EcM-GDH (Initial bacterial concentration 10^7^ ​CFU/ml) assayed at indicated time points. **g,** The glucose concentrations of RPMI 1640 medium treated with EcM-WT or EcM-GDH (Initial bacterial concentration 10^7^ ​CFU/ml) at indicated time point. Data are expressed as means ​± ​s.d. (n ​= ​6, biological replicates).

**Construction and characterizations of a transgenic *E. coli* strain expressing glucose dehydrogenase (EcM-GDH).** The homotetramer GDH is a glucose-depleting enzyme (112.8 ​kDa) that catalyzes the chemical reaction between d-glucose and NAD ​+ ​to consume the glucose substrate with high kinetics. To engineer a microbial strain that efficiently consumes glucose, we proposed introducing the GDH expression system into the microbe. We first placed the GDH gene from *Bacillus subtilis* (sequence number: WP_003246720.1) (Fig. 1a, Table S1) downstream of a constitutive promoter (J23108) in a plasmid with a chloramphenicol resistance gene. After gene cloning and sequencing, the constructed plasmid was then transformed into *E. coli* MG1655 to produce a transgenic bacterium capable of sustained synthesis of GDH protein. We performed the colony PCR to verify whether the GDH gene was transformed into the engineered bacteria. A bright band with a molecular size of 855 bp can be observed on the 1% agarose gel, verifying that the GDH gene has been successfully transformed into *E. coli* MG1655 (Fig. S1). Next, we employed the western blot to characterize the expressed GDH enzyme in the engineered EcM-GDH. As shown in Fig. 1b, a band with a molecular size of 32 ​kDa was only detected in EcM-GDH, consistent with the predicted 31.53 ​kDa GDH protein. These results indicated that the GDH gene had been successfully introduced and produced in the engineered *E. coli*.

The introduction of exogenous genes would likely place a metabolic burden on the host, resulting in the microbe's slow growth or morphological changes. To explore whether the transformation of the GDH expression cassette influences the transgenic *E. coli*, we first compared the proliferation behavior of the engineered strain with that of the WT strain. The results showed that the growth curves of EcM-WT and EcM-GDH largely coincided during the 24 ​h incubation (Fig. 1c). No significant difference in growth rate was found between the two strains during the exponential period, indicating that the GDH expression caused little effect on the growth of the engineered bacteria (Fig. 1d). Notably, we noticed that the EcM-GDH possessed a more extended stationary phase than WT before entering the death phase (Fig. 1c), implying that the engineered bacteria could be employed more durably for glucose consumption. Finally, we performed transmission electron microscopy (TEM) imaging, Furrier-Transition infrared (FT-IR) spectroscopy, and Raman spectroscopy of both strains (Fig. S2a, S2b-c). All these results showed that there were almost no morphological and compositional differences between the transgenic EcM-GDH and WT strains, demonstrating that the constitutively expressed GDH enzyme does not affect the bacteria.

To investigate the glucose consumption capacity of the engineered microbes, we employed a glucose-sensitive chromogenic assay kit (o-toluidine) for glucose concentration monitoring (Fig. 1e, S3a-b). EcM-WT and EcM-GDH were suspended in RPMI-1640 medium (containing 11.11 ​mM of d-glucose) under the same culturing conditions. Aliquots of bacterial cultures (1 ​× ​10^7^ ​CFU/ml) were collected for glucose concentration assay within 6 ​h at 15 ​min intervals. As shown in Fig. 1f, few blue-green products appeared in the bacterial cultures of EcM-GDH at 240 ​min compared to the wide-type strain, demonstrating that EcM-GDH exhibits greater glucose consumption capacities. By linearly fitting the glucose consumption curve, we calculated the glucose consumption rate of the EcM-WT strain to be 25.8 ​μM/min (Fig. 1g). In contrast, the engineered EcM-GDH strain had a 36.9 ​μM/min consumption rate, approximately 1.39-folds of the consumption rate for EcM-WT, signifying that the engineered strains expressing GDH have a more prominent glucose consumption capacity to accomplish local nutrient deprivation for tumor starvation therapy.

**Fig. 2:**
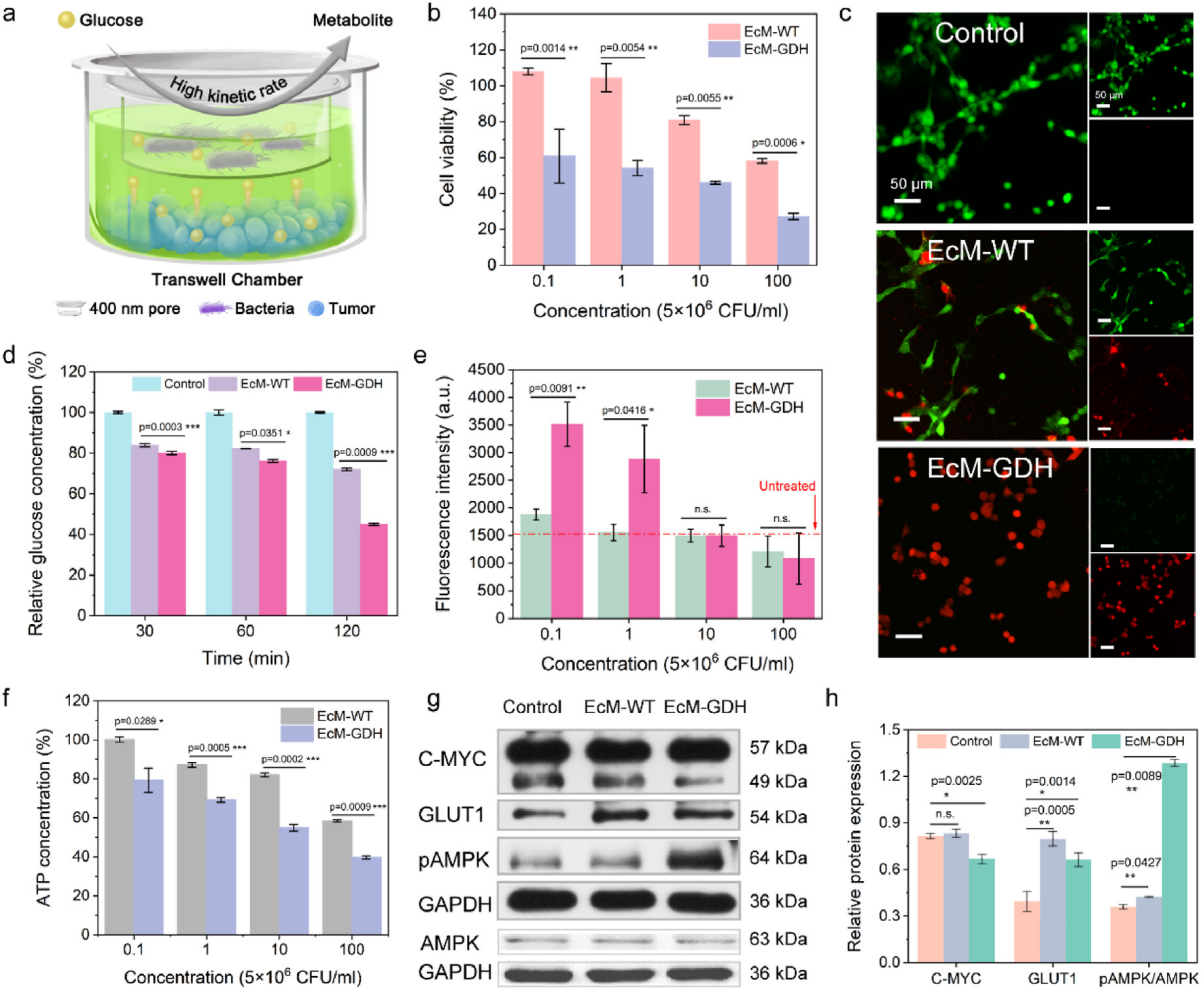
a, Schematic illustration of transwell setup for *in vitro* glucose deprivation evaluation. Bacteria were added into the upper chamber of the transwell while CT26 tumor cells were inoculated into the lower chamber. **b,** Cell viability assays of CT26 ​cells after indicated treatments for 24 ​h. Data are expressed as mean ​± ​s.d. (n ​= ​6, biological replicates). **c,** Confocal microscopic images of calcein-AM (green fluorescence) and PI (red fluorescence) stained CT26 ​cells with indicated treatments. **d,** Relative glucose concentrations in the medium at different time points of the co-incubation system containing CT26 ​cells and EcM-WT or EcM-GDH. Data are presented as mean ​± ​s.d. (n ​= ​6, biological replicates). **e,** Fluorescence intensity profile for cellular uptake assays of cells with indicated treatments. Data are expressed as mean ​± ​s.d. (n ​= ​6, biological replicates). **f,** Relative intracellular ATP concentrations in CT26 ​cells after indicated treatments with varied doses of EcM-WT or EcM-GDH. Data are expressed as mean ​± ​s.d. (n ​= ​6, biological replicates). **g,** Protein expressions of *C*-MYC, GLUT1, pAMPK and AMPK for cells with indicated treatments. **h,** Band quantification of protein expressions. Data are expressed as means ​± ​SD (n ​= ​3). Significances were evaluated by student's t-test, ∗P ​< ​0.05, ∗∗P ​< ​0.01, ∗∗∗P ​< ​0.001 and n. s.

**Intracellular starvation induced by EcM-GDH.** Based on the glucose consumption capability, we next investigated the *in vitro* cellular starvation induction and killing performance by the engineered EcM-GDH bacteria against murine CT26 ​cells (Murine colon carcinoma cell line). A Transwell setup (with a cut-off size of 0.4 ​μm) was employed to isolate the bacterial and the tumor cells during the evaluation (Fig. 2a). With a standard Cell Counting Kit-8 assay, the viability of CT26 decreases along with the increased co-incubation EcM-WT and EcM-GDH dose, presenting dose-dependence cell killing performance originating from glucose-consuming starvation therapy (Fig. 2b). Interestingly, tumor cells treated with EcM-GDH show much higher cytotoxicity (cell viability of 60.7% or 54.1%) at a low bacterial dosage of 5 ​× ​10^5^ ​CFU/ml or 5 ​× ​10^6^ ​CFU/ml respectively, while cells treated with EcM-WT exhibit no observable cytotoxicity at identical bacterial concentration. These profiles demonstrate that the engineered EcM-GDH bacteria could induce a higher cell-killing effect against the tumor cells based on glucose depletion. Specifically, the cell viability of EcM-GDH (5 ​× ​10^8^ ​CFU/ml) was measured at 27.1%, much lower than that of EcM-WT (5 ​× ​10^8^ ​CFU/ml, cell viability of 58.1%), implying their potential to induce lethal starvation against the tumor. The confocal fluorescence microscope was employed to observe Calcein-AM/PI dual-stained cells after respective treatments to visualize the distribution of the live and dead cells subjected to different treatments. It could be observed that a majority of the cells in the control group were stained with green fluorescence (Fig. 2c). When these cells were treated with EcM-WT, few cells were stained with red fluorescence. A majority of the red fluorescence staining cells could be observed for the microscopic images in the EcM-GDH treatment group.

To investigate the molecular mechanism of tumor killing by EcM-GDH, we initially detected the glucose concentration of the medium in the lower chamber of the setup at predetermined time points of 30 ​min, 60 ​min, and 120 ​min (Fig. 2d). We found that the glucose concentration decreases along with the co-incubation time of the tumor cells and bacterial cells. This glucose consumption profile of EcM-GDH remains relatively lower as compared to that of the medium treated with EcM-WT. At t ​= ​120 ​min, the relative glucose concentration of the medium treated with EcM-GDH is calculated as 44.9%, while for the medium treated with EcM-WT, this value is 72.0%, indicating that EcM-GDH could specifically consume glucose nutrients in a faster kinetic to compete for the growth of tumor cells. Moreover, glucose uptake capacity profiles of the tumor cells are monitored using fluorescent, a d-glucose analog (Glucose Uptake Probe-Green. Dojindo) that could be internalized by the cells. After co-incubating EcM-WT or EcM-GDH for 12 ​h, the glucose-containing medium was replaced with a fluorescent d-glucose analog, following the manufacturer's instructions. The intracellular glucose consumption capacity of the cells could then be assessed using the fluorescent microplate reader (Fig. 2e). At low bacterial co-incubation dose of (5 ​× ​10^5^ ​CFU/ml or 5 ​× ​10^6^ ​CFU/ml), little fluorescence intensity differences could be observed between the control group and the EcM-WT group. For EcM-GDH co-incubation with identical doses, dramatically elevated fluorescent intensity could be observed, confirming that the tumor cells are under nutrient-depriving stress with upregulated glucose uptake response. Nevertheless, it is noted that the fluorescent intensity of the cells treated with the higher dose of EcM-GDH (5 ​× ​10^7^ ​CFU/ml or 5 ​× ​10^8^ ​CFU/ml) declined apparently, which may be attributed to the decreased cell population under the circumstance of nutrient starvation stress. Besides, Adenosine triphosphate (ATP), commonly recognized as the energy currency within diverse cell activities, is also an important indicator of cellular energy and starvation status. The cellular ATP levels are evaluated using ATP Assay Kit (Beyotime). We recorded that both cells treated with EcM-WT or EcM-GDH have a decreased profile of ATP levels in a dose-dependent manner. In addition, relatively lower cellular ATP levels could be observed for cells treated with EcM-GDH compared to those treated with the non-engineered counterpart, suggesting that the GDH-engineered bacterial strain presents a much more effective nutrient deprivation capability (Fig. 2f). When the cells were co-incubated with EcM-GDH cells of the highest dose (5 ​× ​10^8^ ​CFU/ml), the cellular ATP levels specifically dropped to 39.6%.

To investigate the molecular axis from glucose depletion to cell death, immunoblot analyses on metabolism-associated proteins were conducted in CT26 ​cells (Fig. 2g and h). The results show that the expression level of MYC, a marker of glycolytic flux, is reduced in cells of the EcM-GDH group, suggesting reduced intracellular glycolytic activity [17]. Comparatively, the expressions of dominant glucose transporter GLUT1 are upregulated in the cells of the EcM-WT group and EcM-GDH group, validating the cell response against glucose depletion – compensating the glycolysis. The GLUT1 expression level of EcM-GDH was lower than that of EcM-WT, which may be attributed to the nutrition deprivation performance of EcM-GDH against the tumor cells. AMPK, a cellular energy status sensor, is able to be phosphorylated under stress or low energy conditions. Such phosphorylation could elevate intracellular ATP levels through the regulation of various metabolic pathways, including increased glucose uptake and autophagy, to compensate for cellular energy stress. The protein expression of phosphorylated AMPK (pAMPK)/AMPK of the EcM-WT group is slightly increased, while the highest expression is observed for the protein expression in the EcM-GDH group, revealing that CT26 ​cells are under strong metabolic stresses originating from the intense glucose depletion enabled by EcM-GDH. Collectively, GDH-engineered *E. coli MG1*655 has been validated to substantially intensify the glucose depletion within tumor cells, decrease cellular glucose uptake and ATP production, impede the glycolytic pathway and activate pAMPK-related energy compensatory mechanism within tumor cells.

**Fig. 3:**
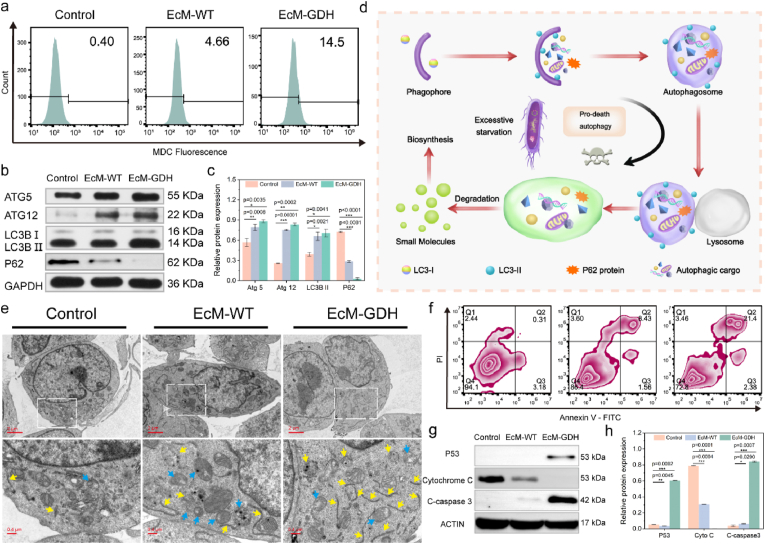
a, Flow cytometric analyses of MDC-stained cells with indicated treatments. **b-c,** Western blot analyses (**b**) and corresponding band quantitative data (**c**) of proteins (ATG5, ATG12, LC3B and p62) in CT26 ​cells after indicated treatments for 8 ​h ​**d,** Schematic illustration of pro-death autophagy mechanism induced by glucose deprivation by EcM-GDH. **e,** Bio-TEM images of CT26 ​cells after co-incubation with different bacteria (upper panel: low magnification; lower panel: high magnification). Blue and yellow arrows indicate the initial and degradative autophagic vacuoles respectively. **f,** Flow cytometric investigation of Annexin V-FITC/PI-strained CT26 ​cells with indicated treatments. **g-h,** Western blot analyses (**g**) and corresponding band quantitative data (**h**) of proteins (p53, Cytochrome C and cleaved-caspase 3) in CT26 ​cells after indicated treatments for 12 ​h (n ​= ​3, biological replicates). Statistical significances were calculated via Student's t-test. ∗P ​< ​0.05, ∗∗P ​< ​0.01, and ∗∗∗P ​< ​0.001.

**In vitro mechanistic evaluation.** It has been documented that cells may perform major cellular alterations in response to nutrient restriction or depletion. It is of significance to gain deeper insights into cellular responses, especially the *in vitro* cell death mechanism. As nutrient starvation is closely associated with autophagy, autophagic marker organelle and autophagic vacuoles were detected by monodansylcadaverine (MDC), a phospholipid-specific fluorescence probe that selectively accumulates inside autophagosomes, using confocal microscopy (Fig. S4a). Cells treated with EcM-GDH exhibit the brightest green fluorescence among the control and other treatment groups. According to the quantitative analyses of confocal microscopy images (Fig. S4b), the number of autophagosomes per cell in the EcM-GDH group was nearly 1.8 folds higher than that of the Control or EcM-WT group, preliminarily demonstrating that persistent starvation could induce cell autophagy. We also quantitatively investigated the localized MDC profile by flow cytometry (Fig. 3a). Cells in the EcM-GDH treatment group display the highest activation degree of MDC fluorescence (14.5%), as compared to Control (0.4%) and EcM-WT groups (4.7%), with the foldchanges calculated to be 36.2 and 3.1 respectively (Fig. S5). From microscopic inspections, we found that abundant intracellular autophagic vacuoles are presented within the cells treated with EcM-GDH, manifesting that nutrient restriction status enabled by EcM-GDH could specifically induce autophagy-associated pathological alterations, including the immediate formation of autophagic vesicles. To further investigate the molecular mechanism of the autophagic process, western blotting was conducted to evaluate the expression of autophagy-related proteins such as ATG5, ATG12, LC3B, and p62 (Fig. 3b and c). According to the blotting results, ATG5 and ATG12 were explicitly upregulated in both EcM-WT and EcM-GDH treatment groups, implicating the increased autophagic flux [18]. Consistent with the intracellular MDC profile, LC3B-II, a typical autophagosomal biomarker, was also upregulated in the cells treated with EcM-WT and EcM-GDH, revealing the increasing quantities of the intracellular autophagosome. In addition, as a specific autophagic substrate, p62 proteins are recruited to autophagosomes and degraded in autolysosomes [19]. Intriguingly, p62 was also downregulated in the EcM-GDH groups and EcM-WT group. With a lower expression profile of the EcM-WT group, an increased intact autophagic flux evoked by the intense and persistent energy deprivation of EcM-GDH could be demonstrated. Although the autophagic process could compensate for the endogenous energy supply in response to nutrient/energy stress, intensive autophagy will result in autophagic cell death that is attributed to excessive damages to overload the degradation capacity of lysosomes [20]. The above results reveal that the intense autophagic flux stimulated by cellular starvation could effectively evoke pro-death autophagy, disrupting the cellular energy metabolism by GDH engineering *E. coli* MG1655 (Fig. 3d). To further confirm the presence of pro-death autophagy, cells subjected to different treatments were sectioned for Bio-TEM observation (Fig. 3e). For cells in the control group, an intrinsic autophagic response could be observed. Several initial and degradative autophagic vacuoles (iAV and dAV, respectively) were found in the cells of the EcM-WT group, as indicated by the yellow and blue arrows, respectively. The quantity of these vacuoles dramatically increases for the cells from the EcM-GDH group, validating the upstream autophagy activation and downstream autophagy suppression.

Considering the regulated AMPK phosphorylation and cellular susceptibility to apoptosis and pro-death autophagy, we are encouraged to evaluate cell apoptotic fate furtherly [21]. Initially, CT26 ​cells with different treatments were stained with Annexin V-FITC/propidium iodide (PI) for flow cytometric analysis (Fig. 3f). Compared to the cells from the control group (3.49% for the Annexin V+ population, revealing both early and late apoptosis), cells incubated with EcM-WT exhibited an elevated percentage of apoptotic cells (7.99%). Of note, the highest percentage of the apoptotic population is observed for the cells from the EcM-GDH group (23.78%), preliminarily suggesting the presence of major apoptotic cells in response to the nutrient starvation enabled by EcM-GDH. We then investigated the underlying molecular mechanism of cell apoptosis using immunoblot. As an AMPK downstream target, the expression of the p53 protein from the EcM-GDH group is highly upregulated as compared to the control and EcM-WT groups, implicating the p53-initiated apoptotic pathway for cellular modulations. We also detected the whole-cell Cytochrome C protein expressions in the cells and found that the Cytochrome C level from the EcM-WT group is approximately half of the expression of the control group. For cells from the EcM-GDH group, Cytochrome C is rarely detected, evidencing mitochondria dysfunction and the release of Cytochrome C. We also show that the apoptotic effector protein, cleaved caspase 3 is slightly upregulation in the EcM-WT group, and highly upregulated in the EcM-GDH group, demonstrating the activation of the ultimate cell apoptosis.

***In vivo* biosafety evaluation.** The prominent performance of EcM-GDH in killing tumor cells encourages us to further investigate the therapeutic potential *in vivo*. Prior to the investigation, an address of biosafety concerns is urgently demanded. Hemolysis is a pathology induced by the destruction of the blood cells by the administration of foreign substances. To guarantee the hemocompatibility, bacterial cells of varied doses were incubated with fresh whole blood from rats to evaluate the hemolytic rates (Figure S6a-b). From the experiment result, we found that both EcM-WT and EcM-GDH (at a dose as high as 5 ​× ​10^8^ ​CFU/ml) exert little hemolysis effect (3.9% and 0.8%, respectively) on the blood, confirming the hemocompatibility of the bacterial cells. We then initiate the *in vivo* biosafety evaluation experiment on randomly assigned healthy Balb/c mice (6 weeks old, n ​= ​5) in three different groups: Control (single i. v. Injection of 100 ​μl PBS); EcM-WT group (single i. v. Injection of EcM-WT of 1 ​× ​10^9^ ​CFU/ml) and EcM-GDH group (single i. v. Injection of EcM-GDH of 1 ​× ​10^9^ ​CFU/ml). The bodyweight of mice was recorded every two days during the evaluation period of 30 days. During the evaluation, no significant variation in bodyweight could be observed for different experimental groups compared to the control group, suggesting that these mice were in good health conditions (Fig. S7a). At the end of the evaluation, these mice were sacrificed with their blood drawn from the eyeballs for routine blood tests and blood biochemistry assays. Major organs (heart, liver, spleen, lung, and kidney) of mice were also dissected and harvested for hematoxylin and eosin (H&E) staining for histopathological inspections. Importantly, no significant sign of inflammation or pathological damage to the mice is found. Histological abnormalities were not observed in the heart, liver, spleen, lung, and kidney of mice from different experimental groups (Figs. S7b–c).

**Fig. 4:**
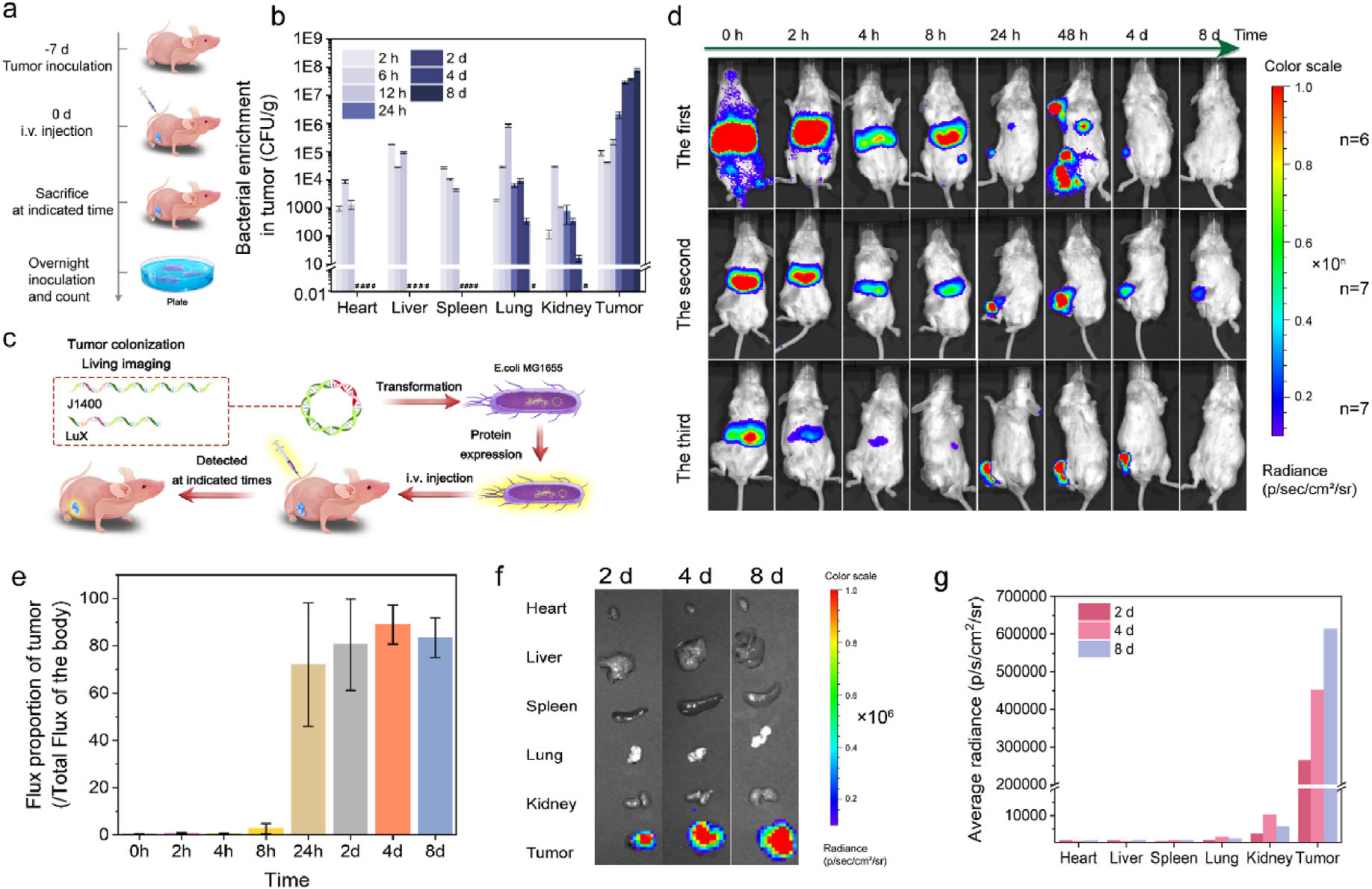
a, Schedule of *in vivo* bacteria distribution and colonization. **b,** Quantification of bacterial colonization in different organs harvested from CT26-bearing mice at different time points after the injection of bacteria. **c,** Schematic illustration of the construction of engineered bacteria expressing chemiluminescence reporter (Lux) for the following intravenous injection and *in vivo* imaging. **d,** In vivo bioluminescence images to track bacterial biodistribution at predetermined time points (0 ​h, 2 ​h, 4 ​h, 8 ​h, 24 ​h, 2 ​d, 4 ​d and 8 ​d) post-injection of EcM-LUX. **e,** The bioluminescence profile from the *in vivo* biodistribution images obtained from Fig. 4d. Data are expressed as mean ​± ​s.d. (n ​= ​3, biological replicates). **f,***Ex vivo* bioluminescence images of the dissected major organs and tumors from mice at indicated time points. **g,** Quantified bioluminescence intensities of the dissected major organs and tumor at indicated time points.

Tumor tropism has been the most advantageous for tumor therapy based on bacteria. The accumulation and retention profile of EcM-GDH towards CT26 tumor-bearing Balb/c mice was monitored upon bacterial administration. Initially, these mice were subcutaneously injected with CT26 ​cells suspended in PBS (1 ​× ​10^6^ ​cells/ml, 100 ​μl) into the right abdomen for subcutaneous colorectal tumor xenograft establishment. When the average volume of xenograft reached ∼200 ​mm^3^, mice were injected with EcM-GDH (dose: 10^9^ ​CFU/ml, 100 ​μl per mouse) intravenously. These mice were euthanized at predetermined timepoints (2 ​h, 6 ​h, 12 ​h, 24 ​h, 2 ​d, 4 ​d, 8 ​d post-i. v. injection) with their major organs (heart, liver, spleen, lung, kidney and tumor) harvested, homogenized and diluted to inoculate the Luria–Bertani (LB) plates for ex vivo bacterial quantitation evaluation (Fig. 4a). At 2 ​h post-injection, bacteria were observed on LB plates treated with the homogenate of main organs and tumor, especially on those of the liver and tumor. The number of bacteria in the major organs increases and maintains to approximately 1 ​× ​10^4^ ​CFU/g in 12 ​h, and reduces gradually over time. Specifically, bacterial clearance from the heart, liver, and spleen was observed at 24 ​h. While for lung and kidneys, bacterial clearance was observed 8 ​d (Figs. S8 and 4b). The results illuminate that the bacterial cells could enrich and colonize selectively inside the tumor tissues, and the bacteria of the main organs are gradually eliminated within 8 ​d. In addition, a reporter stain of EcM was constructed by transforming the Lux-containing plasmid into EcM (EcM-LUX) for constant *in vivo* monitoring at indicated time points (Fig. 4c). Initially, 100 ​μl of varying doses (5 ​× ​10^3^, 5 ​× ​10^4^, 5 ​× ​10^5^, 5 ​× ​10^6^, 5 ​× ​10^7,^ and 5 ​× ​10^8^ ​CFU/ml) of EcM-WT and EcM-LUX were inoculated into the 96-well plate for luminescence observation (Figure S9a-c, S10). Dose-dependent luminescence signals for EcM-LUX could be obtained rather than the non-engineered EcM-WT strain. Next, mice with subcutaneous colorectal tumor xenograft (∼200 ​mm^3^) were injected with EcM-LUX (dose: 100 ​μl, 5 ​× ​10^8^ ​CFU/ml) to evaluate real-time bacterial distribution *in vivo* at predetermined time points (0 ​h, 2 ​h, 4 ​h, 8 ​h, 24 ​h, 48 ​h, 4 ​d and 8 ​d post-administration (Fig. 4d). It can be observed that a major proportion of bacterial cells were enriched to the liver of the mice at 0 ​h, 2 ​h, 4 ​h, 8 ​h post-administration. And then gradually cleared, possibly by the immune system. The percentage of average luminescence intensity of the tumor in the whole body reached 72.1% in 24 ​h and then gradually increased and maintained to 83.4% at 2 ​d, 4 ​d, and 8 ​d. (Fig. 4e). At the end of the evaluation, these mice were sacrificed with their major organs and tumor tissue collected for ex vivo luminescence detection at 2 ​d, 4 ​d, and 8 ​d post-administration (Fig. 4f). From the obtained luminescence images, specifically weak luminescence signals could be observed for major organs, including the heart, liver, spleen, lung, and kidney. For tumor tissue, the luminescence emerged at 2 ​d (263,500 p/s/cm^2^/sr) and persistently increased to 451,500 p/s/cm^2^/sr in 4 ​d and 612,600 p/s/cm^2^/sr in 8 ​d. The statistical data indicate that the average radiance of the major organs is approximately two orders of magnitude lower than that of the tumor tissue. The tumor-specific accumulation and retention profile is attributed to the intrinsic immunosuppression and hypoxia tumor microenvironment that are habitable for the colonization of bacterial cells (Fig. 4g). The luminescence ratios of tumor/liver were calculated by the collected major organs and tumor tissue at 2 ​d, 4 ​d, and 8 ​d post-administration (Fig. S11). The ratios increase and maintain to approximately 1000. Such tumor-specific accumulation paradigm could contribute to the high biocompatibility and biosafety against the host, potentiating their therapeutic prospects *in vivo*.

**Fig. 5:**
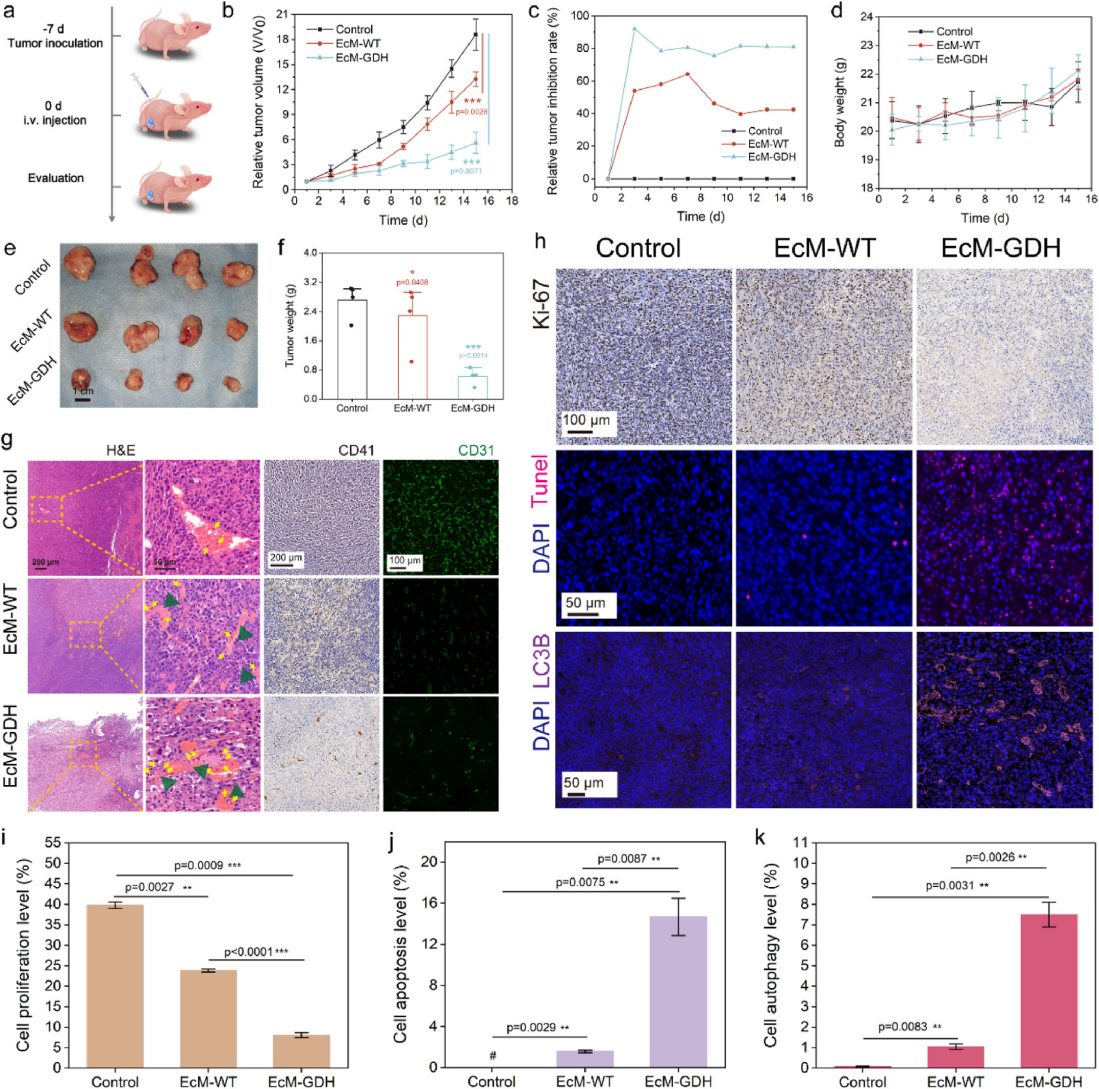
a, Schedule of *in vivo* tumor therapeutic evaluation. **b,** Time-course growth curves of CT26 tumor xenografts in mice from different treatment groups: (1) Control (PBS), (2) EcM-WT or (3) EcM-GDH. Data are expressed as mean ​± ​s.d. (n ​= ​4, biological replicates). Statistical significances were calculated via Student's t-test. ∗P ​< ​0.05, ∗∗P ​< ​0.01, and ∗∗∗P ​< ​0.001. n. s., not significant. **c,** Relative tumor inhibition rate of mice from different treatment groups (n ​= ​4, biological replicates). **d,** Body weight curve of CT26-tumor-bearing mice from different treatment groups during the evaluation period. Data are expressed as mean ​± ​s.d. (n ​= ​4, biological replicates). **e-f,** Photographs (**e**) and weights (**f**) of dissected tumors of mice from different treatment groups. **g,** Microscopic images of H&E stained, CD41 stained, and CD31 immunofluorescence labeled CT26 xenograft tumor sections of mice from different treatment groups at the end of the evaluation period (n ​= ​4, biological replicates). **h**, Microscopic images antigen Ki67 stained or TUNEL, LC3B stained CT26 tumor tissue sections of mice from different treatment groups at the end of the evaluation period (n ​= ​4, biological replicates). **i-k**, Quantifications of Ki67 (**i**), TUNEL (**j**), and LC3B (**k**)-positive nuclei from the images. Statistical significances were calculated via Student's t-test. ∗P ​< ​0.05, ∗∗P ​< ​0.01, and ∗∗∗P ​< ​0.001. n. s., not significant. N ​= ​3, biological replicates.

***In vivo* therapeutics against subcutaneous colorectal tumor by EcM-GDH.** Based on tumor-tropism results and the excellent biosafety performance of EcM-GDH, their *in vivo* therapeutic effects were further evaluated in CT26 tumor-bearing Balb/c nude mice. Twelve mice were randomly allocated into three groups (n ​= ​4): Control, EcM-WT and EcM-GDH groups. These mice were subcutaneously injected with CT26 ​cells suspended in PBS (1 ​× ​10^6^ ​cells/ml, 100 ​μl) into the right abdomen for subcutaneous colorectal tumor xenograft establishment. After 7 days (tumor xenograft reached ∼150 ​mm^3^), the mice from control, EcM-WT and EcM-GDH groups were intravenously administrated with 100 ​μl sterile PBS, EcM-WT of 5 ​× ​10^8^ ​CFU/ml or EcM-GDH of 5 ​× ​10^8^ ​CFU/ml respectively. Mice were weighed, and the tumor size was measured every two days during the evaluation period of 15 days (Fig. 5a). During the therapeutic evaluation, the rapid growth of the tumor xenografts could be observed for the mice in the control groups, while the growth of tumors in the EcM-WT groups was slightly inhibited (Fig. 5b and S12). Importantly, tumors of mice receiving EcM-GDH administration were suppressed to the largest extent. We also calculated each group's relative tumor inhibition (RTI) ratio according to the tumor dimension development curves (Fig. 5c). The RTI ratios of all treatment groups increased at day 3 (53.9% for the EcM-WT group, 92.0% for the EcM-GDH group). For the EcM-WT group, the RTI ratio gradually decreased to 42.4%, while the RTI ratio of EcM-GDH maintained to above 80% from day 3 to day 15. These results indicate that EcM-GDH could effectively inhibit the rapid growth of CT26 tumor *in vivo*, implicating that under the nutrient competition by EcM-GDH bacterial cells, tumor cells were effectively killed under prominent starvation therapy. During the evaluation, no significant weight loss was observed in the treatment groups compared to the control group, confirming negligible adverse effects from these treatments (Fig. 5d). At the end of the evaluation period, all mice were sacrificed, and the major organs and tumor tissues were harvested for further weighting and histopathological analyses. The dissected tumor tissues of mice from different groups clearly indicate the therapeutic consequence of EcM-GDH bacterial starvation therapy (Fig. 5e). According to the weighting results, the xenograft of mice treated with EcM-GDH exhibited the least average weight among the mice in all groups, implicating the most reliable therapeutic strategy enabled by EcM-GDH (Fig. 5f).

To further confirm the therapeutic mechanism responsible for therapeutic effects, H&E-stained tumor sections were inspected. EcM-GDH displayed fewer tumor cell nuclei than those of the other treatment groups (Fig. 5g). In addition, the neutrophil invasion could be found in tumor vessels for EcM-WT and EcM-GDH groups rather than the control group, as indicated by yellow arrows. Thrombosis was observed in EcM-WT and EcM-GDH groups as the fibrin-containing clots. To further validate tumor thrombosis after bacteria injection, CD41, a typical marker of activated platelets receptor for soluble fibrinogen and fibronectin, was conducted for immunohistochemical staining of the tumor section. From microscopic images of CD41-labeled tumor sections, higher quantities of platelets could be found in the sections of EcM-WT and EcM-GDH groups, illuminating that both EcM-WT and EcM-GDH bacterial cells could effectively trigger localized tumor-thrombosis (Fig. 5k). Considering the association between bacteria retention and colonization through tumor vessels, CD31, a marker of blood vascular endothelial, was employed for immunofluorescent staining of the tumor sections (Fig. 5k). Tumor sections from the control group displayed abundant CD31 fluorescence, while CD31-labeled vessels from the EcM-WT and EcM-GDH were sparse. This comparison indicates that the *E. coli* MG1655 harbors a potent performance of anti-angiogenesis and neovasculature obstruction and further provokes neutrophil invasions and triggers tumor-specific thrombosis rather than other major organs. Such thrombosis circumstance was also observed in other works reported previously [7a]. In addition, such tumor-specific thrombus formation enabled by EcM-GDH could further impede exogenous nutrient supply to tumor tissues, resulting in an enhanced starvation therapeutic effect.

In addition, antigen Ki67, TdT-mediated dUTP nick-end labeling (TUNEL), and LC3B immunostaining of tumor tissue sections show that EcM-GDH has the least Ki67-positive cells and the most TUNEL- and LC3B- positive cells among various treatments (Fig. 5h–k), further revealing the effective induction of apoptosis and pro-death autophagy against the malignant tumor burden. The present paradigm is specifically beneficial to induce targeted apoptosis and pro-death autophagy against malignant tumors through nutrient deprivation and starvation induction with high biocompatibility.

## 3 Conclusion

In summary, we have successfully constructed a catalytic nutrient depriver by introducing the GDH enzyme synthetic pathway into the non-pathogenic *E. coli* MG1655. Compared to the wild-type strain, the genetically engineered EcM-GDH strain exhibit a 1.39-fold increase in glucose consumption capability, as demonstrated in the *in vitro* glucose consumption test. Given the nutrition competition relationships between bacteria and tumor cells, the EcM-GDH strain can effectively diminish the intracellular glycolytic flux and disrupt the mitochondrial hemostasis of tumors both *in vitro* and *in vivo*, activating the pro-death autophagy and p53-initiated apoptosis pathways in response to the catalytic nutrient deprivation. The engineered EcM-GDH bacterial cells can specifically accumulate inside the tumor tissue with a tumor/liver distribution ratio of 1172.7 ​at 4 ​d post-administration. These engineered microorganisms exhibit significant anti-tumor performance at a dose of 1 ​× ​10^7^ ​CFU/mice administered intravenously, reducing the colorectal subcutaneous tumor burden by above 80%. The immunohistochemical and immunofluorescence inspections demonstrate significant local tumor thrombosis due to the intratumoral bacterial enrichment, enhancing vascular blockade and nutrient restriction. In conclusion, the present paradigm in creating genetically engineered tumor-targeting microbes for catalytic nutrient deprivation is of appealing potential, especially in metabolic regulation-based tumor starvation therapy with high biocompatibility.

## 4 Methods

### 4.1 Material and reagents

All gene information came from National Center for Biotechnology Information (USA), and all genes were synthesized by GENEWIZ (New Jersey, USA) and then amplified by polymerase chain reaction (PCR). The *E. coli* MG1655 and *E. coli* DH5α strains were bought from Beijing TransGen Biotech Co., Ltd. (Beijing, China). By Sangon Biological Engineering Technology (Shanghai, China), Luria-Bertani (LB) broth medium and LB agar medium was purchased, and oligo primers were synthesized. Glucose Assay Kit with O-toluidine, ATP assay kit, Autophagy Staining Assay Kit with MDC, and 4,6-diamidino-2-phenylindole (DAPI) was obtained from Beyotime Biotech Institute (Shanghai, China). Cell Counting Kit-8 (CCK-8) and Annexin V-FITC/PI were obtained from Dojindo Molecular Technologies. Anti-C-MYC (Cat#. ab32072), Anti-GLUT1 (Cat#. ab115730), Anti-pAMPK (Cat#. ab133448), Anti-AMPK (Cat#. ab32047), Anti-ATG5 (Cat#. ab108327), Anti-p62 (Cat#. ab109012), Anti-p53 (Cat#. ab26), Anti-Cytochrome C (Cat#. ab133504), Anti-Cleaved-caspase 3 (Cat#. ab214430), Anti-CD31(Cat#. ab182981) and Anti-CD41 (Cat#. ab134131) were obtained from Abcam. Anti-LC3B (Cat#. 43,566) was obtained from CST. Anti-ATG12 (Cat#.11122-1-AP) was obtained from Proteintech. Trans DNA Marker I and DNA 1 ​kb DNA Ladder were purchased from TransGen Biotech Co., Ltd. (Beijing, China). All reagents were used without further purification.

**Characterizations.** Transmission electron microscopy (TEM) photographs were obtained by a JEM-2100 ​F electron microscope (JEOL, Tokyo, Japan) operated at 200 ​kV. Sanger sequencing was performed at Sangon Biological Engineering Technology (Shanghai, China). FTIR spectra were acquired on Nicolet iS 10 (Thermo Scientific, Waltham, Massachusetts). Confocal laser scanning microscopy (CLSM) images were recorded on FV1000 (Olympus Company, Japan). Absorbance and luminescence profiles were recorded using the microplate reader (Biotek Instruments, Inc., Winooski, VT, USA). Flow cytometric analyses were performed using BD LSRFortessa flow cytometry (BD, USA). The *in vivo* fluorescence images were acquired using IVIS Lumina (PerkinElmer, USA) and were analyzed by In Vivo Imaging Software.

**Strain construction.** GDH or Lux genes were amplified by PCR with primers. The purified PCR product was integrated into the expression plasmid by Gibson assembly and then transformed into *E. coli* DH5α. LB agar plates containing 34 ​μg/ml chloramphenicol were used to screen bacteria with the transformed plasmids. Primers GDH-Forward, GDH-Reverse, Lux-Forward, and Lux-Reverse were used to perform colony PCR experiments for GDH and Lux, respectively (Table S2). Selected microbial colonies were sent for Sanger sequencing. After sequencing, the plasmids with the correct sequence were transformed into *E. coli MG1655* to obtain the strains of EcM-GDH and EcM-LUX.

To characterize the Lux protein expression, bacteria that harbored the Lux gene were cultured in 96-well plates for luminescence detection using the living imaging system spectrum.

**Bacterial proliferation and quantification assay.** Bacteria (EcM-WT, EcM-GDH, and EcM-LUX) at different initial concentrations (5 ​× ​10^5^ and 5 ​× ​10^6^ ​CFU/ml) were added into transwell 24 -well plates, and O.D. 600 was measured using microplate readers over 24 ​h at the interval of 15 ​min.

**Glucose consumption assay.** EcM-WT and EcM-GDH were incubated in 3 ​ml RPMI 1640 medium (initial glucose concentration: 11.11 ​mM, initial bacterial concentration: 5 ​× ​10^7^ ​CFU/ml) at 37 ​°C, respectively. During the evaluation period of 240 ​min, aliquots of the medium were extracted from the assay solution at 15 ​min intervals for glucose analysis using Glucose Assay Kit (Beyotime).

**Cell experiments.** Murine colon carcinoma CT26 ​cell line was obtained from the Cell Bank Type Culture Collection of the Chinese Academy of Sciences (Shanghai, China). CT26 cells were cultured in RPMI 1640 medium containing 10% fetal bovine serum in a humidified incubator under 37 ​°C, supplied with 5% CO_2_.

**Cell viability assay.** CT26 cells were seeded in the lower chamber of transwell 24-well plate containing RPMI 1640 medium and incubated overnight (initial density: 5 ​× ​10^4^ ​cells per well). After cell attachment, the medium was discarded, and the cells were rinsed three times, followed by the replacement of 500 ​μl of fresh medium. Another 500 ​μl of fresh medium containing varying doses of EcM-WT or EcM-GDH (1 ​× ​10^6^, 1 ​× ​10^7^, 1 ​× ​10^8^, and 1 ​× ​10^9^ ​CFU/ml) were added into the upper chamber respectively. CCK-8 assay was performed in 24 ​h co-incubation.

**Confocal Microscopic Imaging.** CT26 cells were seeded at 1 ​× ​10^6^ ​cells per well in the confocal-exclusive transwell 6-well plate containing RPMI 1640 medium and incubated overnight. After cell attachment, the medium was discarded, and the cells were rinsed three times, followed by the replacement of 1 ​ml of fresh medium. Another 1 ​ml of fresh medium containing EcM-WT or EcM-GDH (final concentration: 1 ​× ​10^8^ ​CFU/ml) were added into the upper chamber. In 24 ​h, cells were rinsed and stained with Calcein-AM (Ex: 488 ​nm, Em: 515 ​nm) and PI (Ex: 559 ​nm, Em: 619 ​nm) dyes, followed by confocal observations. All experiments were repeated three times.

**In vitro glucose consumption assay.** CT26 cells were seeded in the lower chamber of transwell 6-well plate containing RPMI 1640 medium and incubated overnight (initial density: 1 ​× ​10^6^ ​cells per well). After cell attachment, the medium was discarded, and the cells were rinsed three times, followed by the replacement of 1 ​ml of fresh medium. Another 1 ​ml of fresh medium containing varying doses of EcM-WT or EcM-GDH (1 ​× ​10^6^, 1 ​× ​10^7^, 1 ​× ​10^8^ and 1 ​× ​10^9^ ​CFU/ml) were added into the upper chamber respectively. At predetermined time points (30 ​min, 60 ​min, 120 ​min), aliquots of the solution in the lower chamber were extracted from the assay solution for glucose concentration determination.

**Glucose uptake assay.** CT26 cells were seeded in the lower chamber of transwell 24-well plate containing RPMI 1640 medium and incubated overnight (initial density: 5 ​× ​10^4^ ​cells per well). After cell attachment, the medium was discarded and the cells were rinsed three times, followed by the replacement of 500 ​μl of fresh medium. Another 500 ​μl of fresh medium containing varied doses of EcM-WT or EcM-GDH (1 ​× ​10^6^, 1 ​× ​10^7^, 1 ​× ​10^8^ and 1 ​× ​10^9^ ​CFU/ml) were added into the upper chamber respectively. Then the medium was replaced with the modified medium containing fluorescent glucose analog instead of glucose. In 24 ​h, fluorescence measurements of the cells were performed to evaluate the glucose uptake capacity of tumor cells.

**Western blot.** CT26 cells were seeded in the lower chamber of transwell 6-well plate containing RPMI 1640 medium and incubated overnight (initial density: 1 ​× ​10^6^ ​cells per well). After cell attachment, the medium was discarded and the cells were rinsed three times, followed by the replacement of 1 ​ml of fresh medium. Another 1 ​ml of fresh medium containing varied doses of EcM-WT or EcM-GDH (1 ​× ​10^9^ ​CFU/ml) were added into the upper chamber, respectively. Cells were co-incubated with bacteria for 6 ​h. Afterward, cells were rinsed with cold PBS and collected after trypsin digestion and centrifugation. The collected cells were then treated with RIPA (Radio Immunoprecipitation Assay) lysis buffer and centrifuged at 12,000 ​r.p.m. for protein collection and further quantification through BCA (Bicinchoninic acid) assays. For tissue samples, 50 ​mg of tissue was homogenized and centrifuged. The supernatants were added into the loading buffer and heated at 100 ​°C for 5 ​min followed by immediate transfer to the cold ice. Proteins (20 ​μg) were loaded in the electrophoretic buffer solution and treated at 70 ​V for 30 ​min. After separation, proteins were transferred to polyvinylidene fluoride membrane and blocked with 5% bovine serum for 2 ​h in TBST, followed by primary antibodies (*Anti*-C MYC, Anti-GLUT1, *Anti*-pAMPK, *Anti*-ATG5, *Anti*-ATG12, *Anti*-LC3B, *Anti*-p62 *Anti*-p53, Anti-Cytochrome c, and Anti-Cleaved-caspase 3) co-incubation at 4 ​°C overnight. Then, samples were further incubated with horseradish peroxidase (HRP)-conjugated anti-rabbit IgG and anti-mouse IgG for 2 ​h at room temperature. Finally, electrochemiluminescence bands were obtained after scotography. Grayscale analysis of the protein bands was measured by using Image J Software (version 1.51j8).

**MDC staining.** CT26 cells were seeded in the lower chamber of transwell 6-well plate containing RPMI 1640 medium and incubated overnight (initial density: 1 ​× ​10^6^ ​cells per well). After cell attachment, the medium was discarded and the cells were rinsed three times, followed by the replacement of 1 ​ml of fresh medium. 1 ​ml of another fresh medium containing varied doses of EcM-WT or EcM-GDH (1 ​× ​10^9^ ​CFU/ml) were added into the upper chamber, respectively. In 8 ​h, cells were rinsed and stained with MDC for 30 ​min, followed by confocal microscopic observations (Ex: 335 ​nm, Em: 512 ​nm). The experiments were replicated three times biologically. The number of autophagic vacuoles in different groups was counted using Image J Software (version 1.51j8).

**Flow cytometry.** CT26 cells were seeded in the lower chamber of transwell 6-well plate containing RPMI 1640 medium and incubated overnight (initial density: 1 ​× ​10^6^ ​cells per well). After cell attachment, the medium was discarded and the cells were rinsed three times, followed by the replacement of 1 ​ml of fresh medium. 1 ​ml of another fresh medium containing varied doses of EcM-WT or EcM-GDH (1 ​× ​10^9^ ​CFU/ml) were added into the upper chamber, respectively.

For MDC investigation, after 8 ​h, cells were washed and resuspended in a binding solution containing MDC dye (Ex: 335 ​nm, Em: 512 ​nm) for 30 ​min. Then the cells were rinsed with PBS three times and analyzed using the flow cytometer.

For apoptosis investigation, after 24 ​h, cells were collected and resuspended in Annexin V-FITC (Ex: 494 ​nm, Em: 518 ​nm)/PI (Ex: 535 ​nm, Em: 617 ​nm) stain solution for 30 ​min. Then the cells were rinsed with PBS three times and analyzed using the flow cytometer.

Data were collected using the software CytExpert (version 2.2) and then analyzed via FlowJo (version 10.0). The experiments were replicated three times biologically.

**Bio-TEM.** CT26 cells were seeded in the lower chamber of transwell 6-well plate containing RPMI 1640 medium and incubated overnight (initial density: 1 ​× ​10^6^ ​cells per well). After cell attachment, the medium was discarded and the cells were rinsed three times, followed by the replacement of 1 ​ml of fresh medium. 1 ​ml of another fresh medium containing varied doses of EcM-WT or EcM-GDH (1 ​× ​10^9^ ​CFU/ml) were added into the upper chamber respectively. In 6 ​h, these cells were collected using a cell scraper, fixed, and sectioned to prepare ultrathin slices for autophagic vacuole observation (JEM-2100 ​F electron microscope). Then the cells were rinsed with PBS three times and analyzed using a flow cytometer. The data were collected via the software CytExpert (version 2.2) and then analyzed via FlowJo (version 10.0). All experiments were repeated three times.

**Animal Experiments.** Female Sprague-Dawley rats, ICR mice, BALB/c mice and BALB/c nude mice were obtained from Shanghai Laboratory Animal Center. All animal studies conform to the guidelines of the Animal Care Ethics Commission of Shanghai Tenth People's Hospital (SHDSYY-2020-Z0026). Mice were housed in ventilated stainless-steel cages under standard conditions (light: 12 ​h light/dark cycle, ambient temperature: 25 ​± ​2 ​°C, humidity: 60 ​± ​10%), with free access to food and water.

**Hemolysis Assay.** Fresh whole-blood samples were collected from the orbital venous of Sprague-Dawley rats. The blood samples were centrifugated at 1000 ​r.p.m. for 15 ​min and gently rinsed with saline to collect erythrocytes. These cells were diluted with saline to 2 ​wt% for further use. In a typical assay, 500 ​μL of the diluted erythrocytes suspension was mixed with 500 ​μL EcM-WT or EcM-GDH suspension (in saline) at varied concentrations (1 ​× ​10^4^, 1 ​× ​10^6^, 1 ​× ​10^6^, 1 ​× ​10^7^, 1 ​× ​10^8^ and 1 ​× ​10^9^ ​CFU/ml). Saline and deionized water were set as negative and positive controls, respectively. The mixed dispersions were incubated at 37 ​°C for 3 ​h and then centrifugated at 10,000 ​g for 15 ​min. Optical absorptions of the supernatant at 540 ​nm were measured by a microplate reader. The hemolysis ratio was calculated according to the following formula:

$$Hemolysis\;rate\;\left(\%\;of\;control\right)=\frac{A_{sample}-A_{saline}}{A_{water}-A_{saline}}\times 100\%$$

***In vivo* biosafety evaluation.** Fifteen female ICR mice (6-weeks old) were randomly divided into five groups: control (untreated), EcM-WT (bacteria dose: 100 ​μl, 1 ​× ​10^9^ ​CFU/ml, single i. v.) and EcM-GDH (bacteria dose: 100 ​μl, 1 ​× ​10^9^ ​CFU/ml, single i. v.) groups. Upon respective administrations, mice were evaluated for 24 days. The body weight of mice was recorded every 2 days. At the end of the experiment, blood of mice was drawn from eyeball for hematological analysis and blood biochemical analysis. Mice were then sacrificed by painless cervical dislocation. The major organs (heart, liver, spleen, lung, and kidney) of mice were harvested for H&E staining.

***In vivo* biodistribution assay.** Female Balb/c mice bearing subcutaneous CT26 murine colon tumor xenografts (∼200 ​mm^3^) were intravenously injected with *E. coli MG1655* at 1 ​× ​10^8^ ​CFU per mouse. Mice were sacrificed at predetermined time points (2 ​h, 6 ​h, 12 ​h, 24 ​h, 2 ​d, 4 ​d, and 8 ​d). The major organs (heart, liver, spleen, lung, and kidney) and tumors with bacterial injection were collected, weighted and homogenized at 4 ​°C in sterile PBS (pH ​= ​7.4). These samples were then diluted (dilution ratio: 1/1000) and inoculated on LB agar plates. In 12 ​h incubation, bacterial colonies were counted. The bacterial accumulation profiles (CFU per gram of tissue) were calculated with colony counts per gram of tissue weight.

For *in vivo* luminescence profiling, female Balb/c mice (6 weeks old) with CT26 tumor xenograft (∼200 ​mm^3^) were injected with EcM-LUX (bacteria dose: 100 ​μl, 1 ​× ​10^9^ ​CFU/ml, single i. v.). *In vivo* luminescence images were recorded in 0 ​h, 2 ​h, 4 ​h, 8 ​h, 24 ​h, 48 ​h, 4 ​d and 8 ​d post-administration using *in vivo* imaging system (IVIS). In 2 ​d, 4 ​d, and 8 ​d post-administration, one of mice from each group was sacrificed for main organ dissection and ex vivo luminescence imaging.

***In vivo* tumor therapeutics.** Twenty 4-week-old female Balb/c mice were allocated randomly into five groups (n ​= ​4) and inoculated with 10^6^ CT26 ​cells per mouse. When the average tumor volume reached ∼150 ​mm^3^, mice were respectively treated with PBS, EcM-WT, or EcM-GDH (bacteria dose: 100 ​μl, 5 ​× ​10^8^ ​CFU/ml, single i. v.) to initiate the therapeutic experiment. During the evaluation period of 15 days, tumor dimensions were measured and the weight of mice were recorded every two days. At the end of the evaluation period, mice were sacrificed and the tumor xenografts were harvested, weighted and photographed. Xenografts were fixed with 4% paraformaldehyde and sectioned for further H&E staining, immunohistochemistry, and immunofluorescence.

The tumor volume was calculated using the formula:

$$V=\frac{a\times b^{2}}{2}$$

where a refers to tumor length and b refers to tumor width, supposing a ≥ b.

## Statistical Analysis

The statistical significances in this work were analyzed via a two-sided Student's t-test using the software SPSS 20 statistics (version 26.0), n. s. For non-significant; ∗P ​< ​0.05; ∗∗P ​< ​0.01; ∗∗∗P ​< ​0.001.

## Credit author statement

**Penghao Ji**: Investigation, Methodology, Validation, Software, Formal analysis, Data curation, Visualization, Writing – original draft. **Bolin An**: Methodology, Resources, Writing – review & editing. **Zhongming Jie**: Investigation. **Liping Wang**: Methodology. **Shuwen Qiu:** Methodology. **Changhao Ge**: Methodology. **Qihui Wu**: Resources. **Jianlin Shi**: Resources, Funding acquisition, Writing – review & editing. **Minfeng Huo**: Conceptualization, Supervision, Funding acquisition, Writing – review & editing.

## Declaration of competing interest

The authors declare that they have no known competing financial interests or personal relationships that could have appeared to influence the work reported in this paper.

## Data Availability

Data will be made available on request.
