# Supplementary material for: Genetically engineered probiotics as catalytic glucose depriver for tumor starvation therapy
Source: Mater Today Bio. 2022 Dec 15;18:100515. doi: 10.1016/j.mtbio.2022.100515 (PMC9792908; doi:10.1016/j.mtbio.2022.100515)
Supplement: Multimedia component 1 [file mmc1.docx]

Supporting Information of

**Genetically Engineered Probiotics as Catalytic Glucose Depriver for Tumor Starvation Therapy**

Penghao Ji^1,2,3^, Bolin An^4,5^, Zhongming Jie^2,6^, Liping Wang^2,3^, Shuwen Qiu^1^, Changhao Ge^4,5^, Qihui Wu^7^, Jianlin Shi^1,2,3^, Minfeng Huo^1,2,3^*

^1^ Shanghai Tenth People's Hospital, Shanghai Frontiers Science Center of Nanocatalytic Medicine, School of Medicine, Tongji University, Shanghai, 200072, P. R. China.

^2^ State Key Laboratory of High Performance Ceramics and Superfine Microstructure, Shanghai Institute of Ceramics Chinese Academy of Sciences; Research Unit of Nanocatalytic Medicine in Specific Therapy for Serious Disease, Chinese Academy of Medical Sciences (2021RU012), Shanghai 200050, P. R. China.

^3^ Center of Materials Science and Optoelectronics Engineering, University of Chinese Academy of Sciences, Beijing, 100049, P. R. China.

^4^ Center for Materials Synthetic Biology, Shenzhen Institute of Synthetic Biology, Shenzhen Institute of Advanced Technology, Chinese Academy of Sciences, Shenzhen 518055, China.

^5^ CAS Key Laboratory of Quantitative Engineering Biology, Materials Synthetic Biology Center, Shenzhen Institute of Synthetic Biology, Shenzhen Institute of Advanced Technology, Chinese Academy of Sciences, Shenzhen, 518055, P. R. China.

^6^ School of Physical Science and Technology, Shanghai Tech University, Shanghai 201210, P.R. China.

^7^ Translational Research Institute of Brain and Brain-Like Intelligence, Shanghai Fourth People’s Hospital Affiliated to Tongji University School of Medicine, Shanghai, 200081, P. R. China.

Corresponding author: Prof. Minfeng Huo (mfhuo@tongji.edu.cn)

**Supplementary Figures**

**
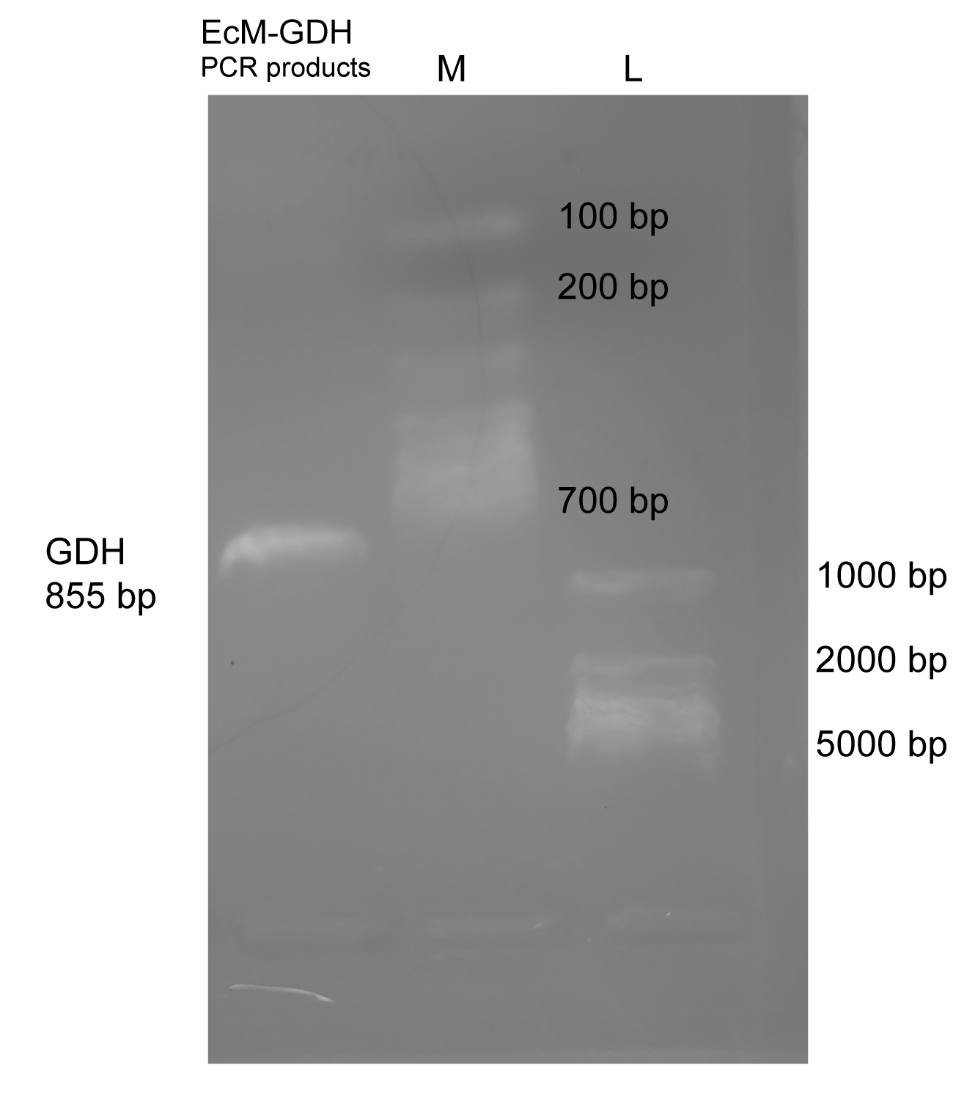
**

**Figure S1.** Agarose gel electrophoresis photograph of PCR products of EcM-GDH.

M: *Trans* DNA Marker I. L: DNA 1kb DNA Ladder.


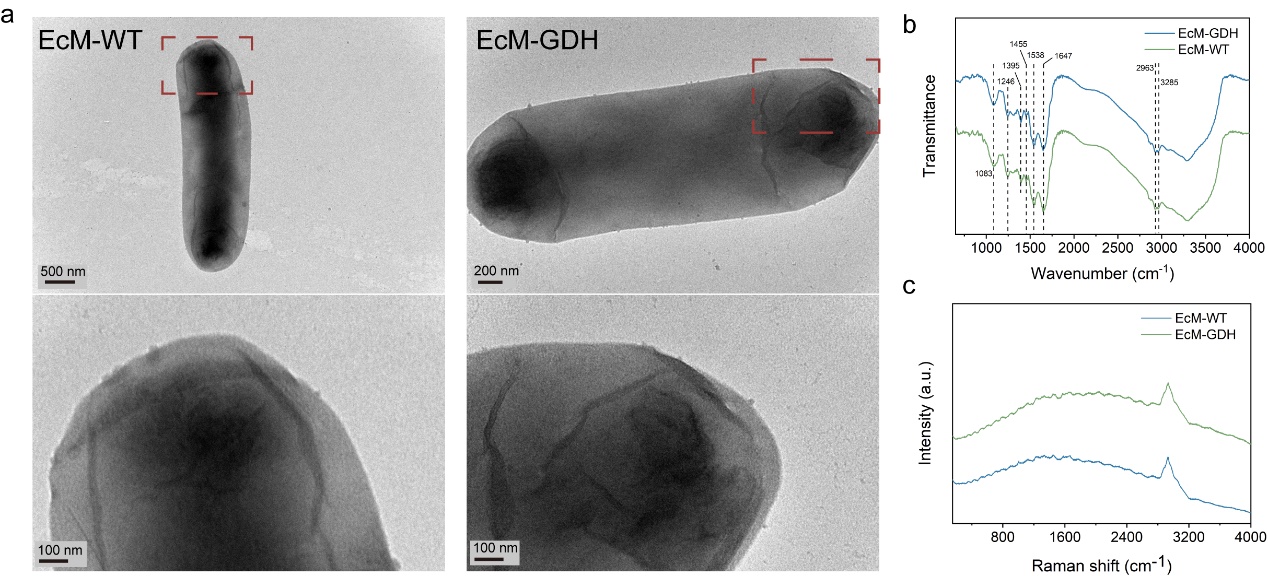


**Figure S2.** **a-b,** TEM images (a), FTIR patterns (**b**), and Raman spectra (**c**) of EcM-WT and EcM-GDH.


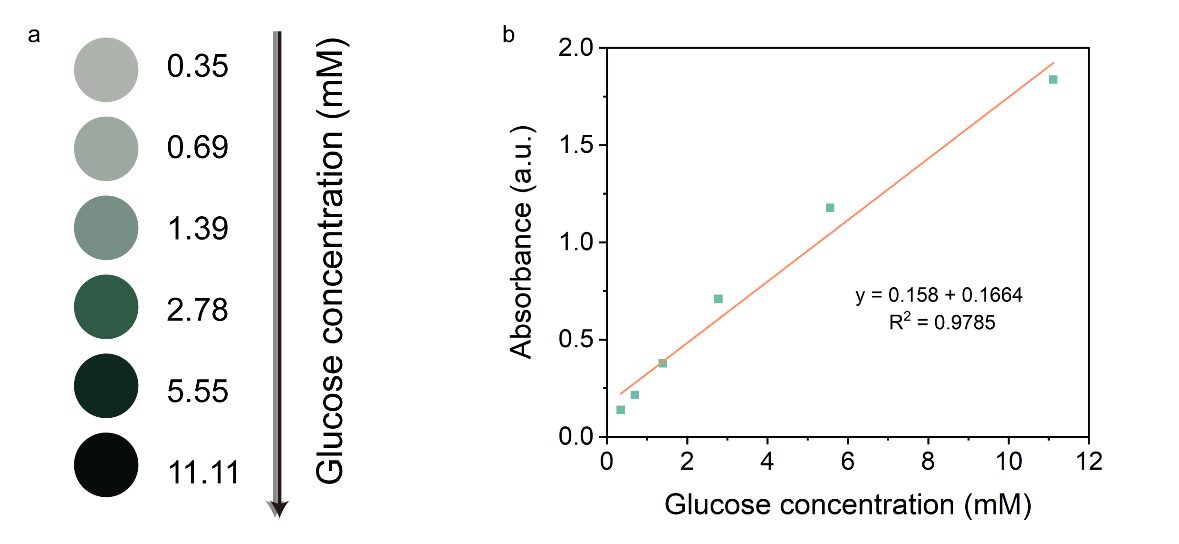


**Figure S3.** **a,** The color of the reaction products between O-toluidine and varied known concentrations of glucose. **b,** Standard curve of varied known concentrations of glucose.


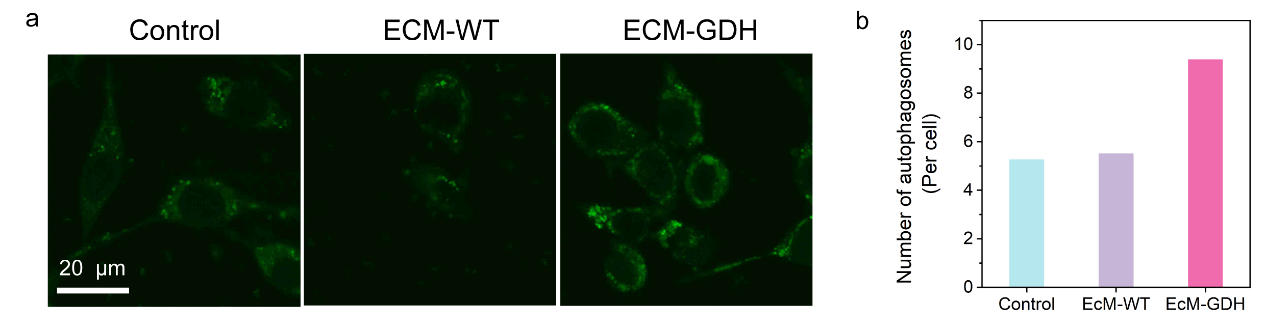


**Figure S4.** **a,** Confocal microscopic images of autophagy levels (cells stained with MDC) after the treatments as indicated above. **b,** Number of autophagosomes per cell in the different treatment groups.





**Figure S5. a,** Quantified fold change of autophagy intensity of EcM-WT and EcM-GDH compared to control.


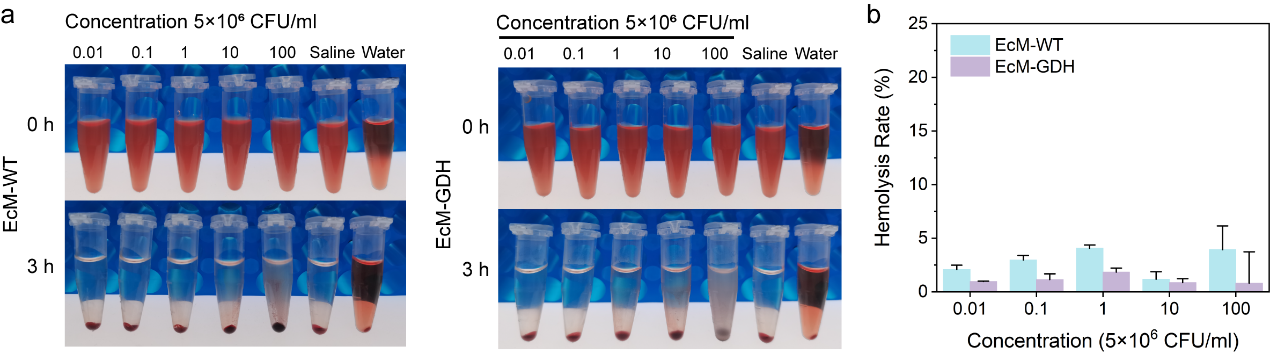


**Figure S6. a-b,** In vitro hemolysis test (photographs (**a**) and corresponding quantification (**b**)) of EcM-WT and EcM-GDH. Saline and water were used as negative and positive controls, respectively. Data are expressed as means ± s.d. (n = 3, biological replicates).


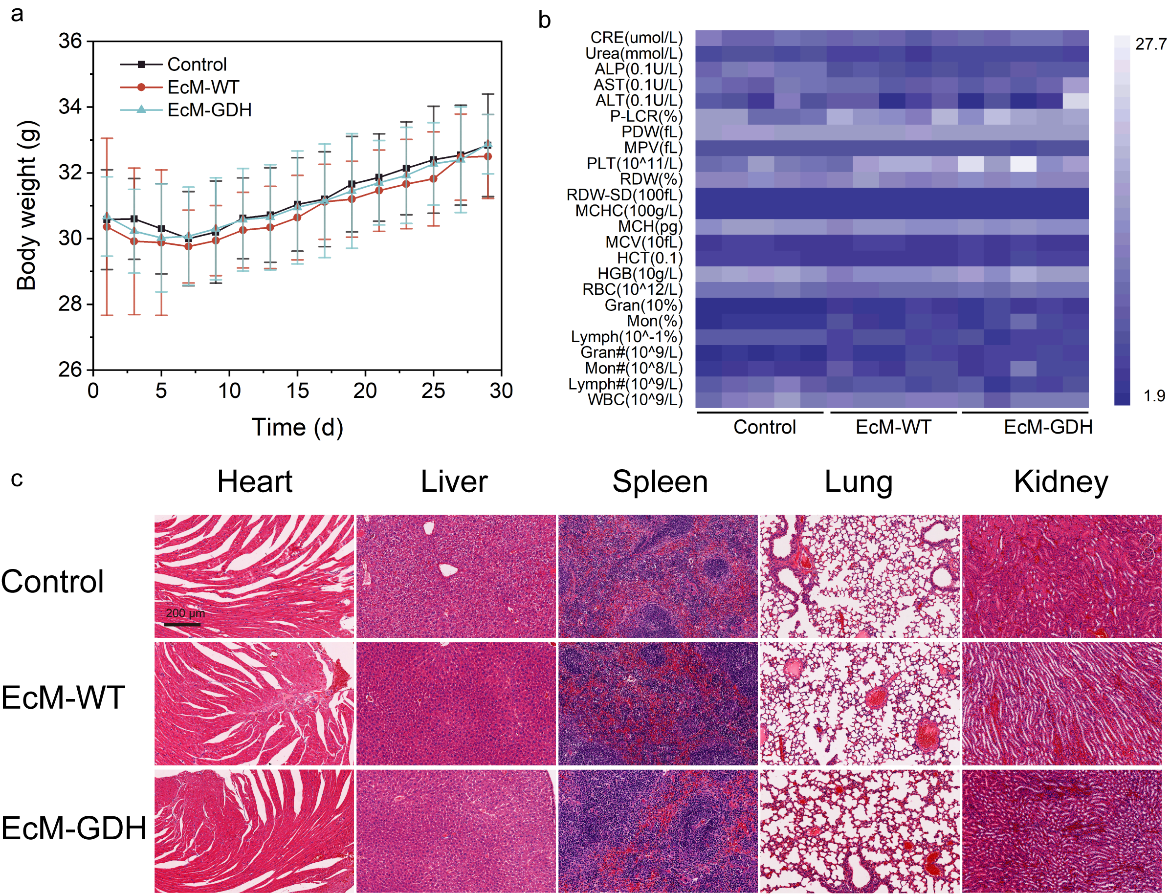


**Figure S7.** **a**, The body weight curves of ICR mice from different groups (n = 5, biological replicates) during the evaluation period of 30 days. Data are presented as mean ± s.d. **b**, The heat maps of both blood biochemical parameters and blood routine index of mice from different groups (n = 5, biological replicates) on day 30. **c**, H&E stained microimages of heart, liver, spleen, lung, and kidney from different treatment groups on day 30.


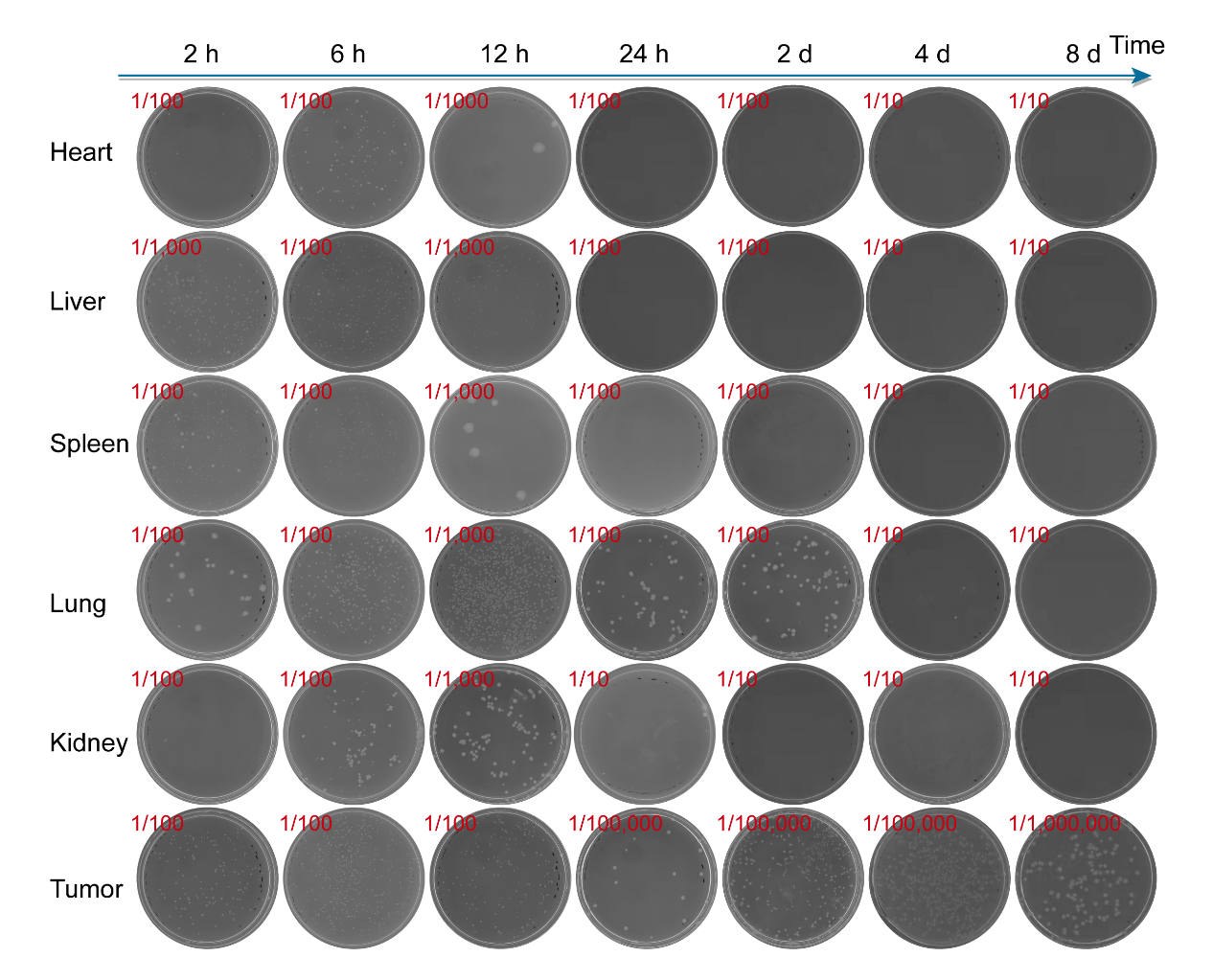


**Figure S8.** Representative photographs of Luria-Bertani (LB) agar plates of bacterial colonization in various organs harvested from CT26-bearing mice at different time points after injection of bacteria.


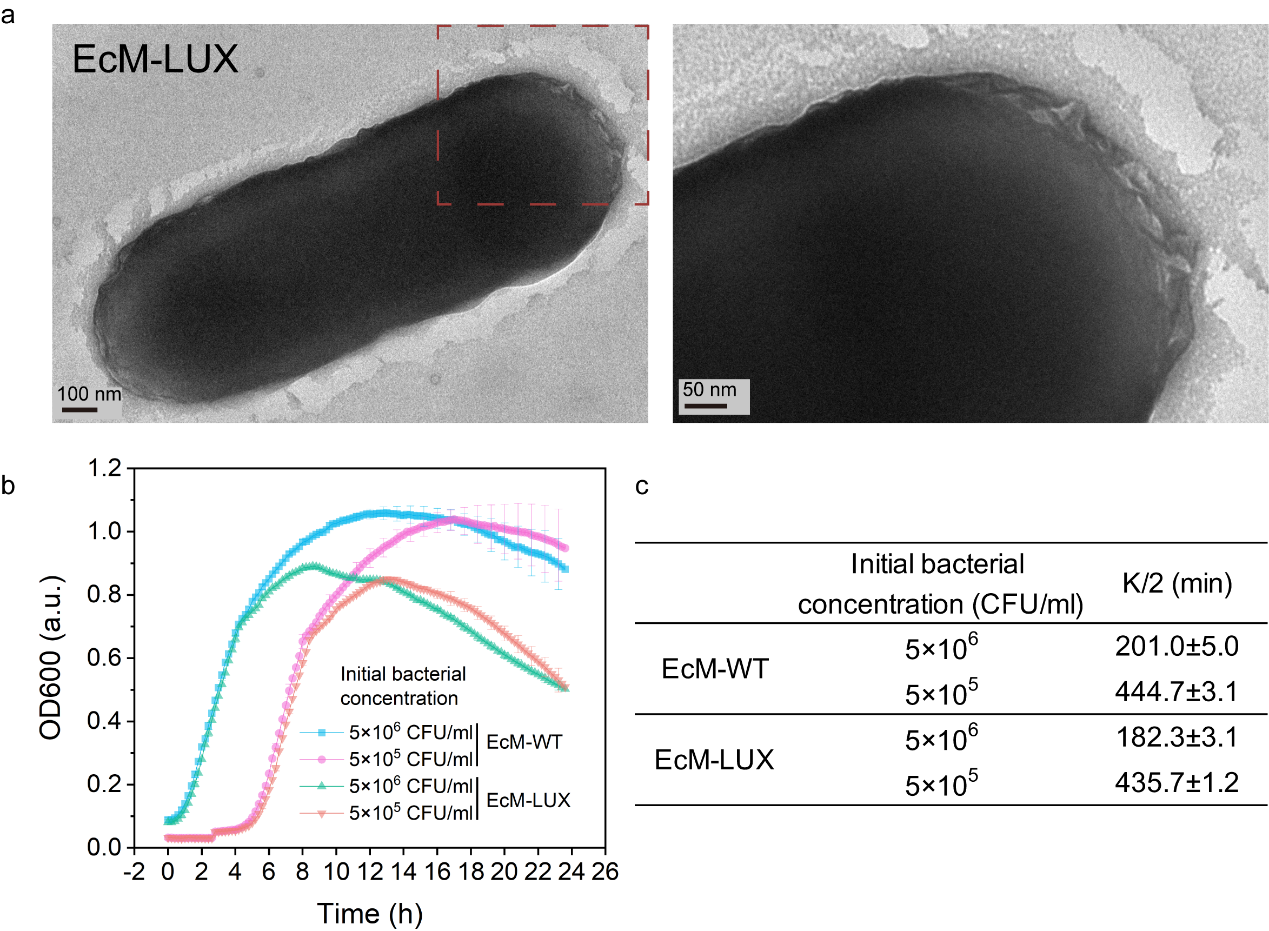


**Figure S9.** **a,** TEM images of EcM-LUX. **b,** The growth curve of EcM-LUX in LB broth medium over 24 h. Data are expressed as means ± s.d. (n = 3, biological replicates). **c,** Time for bacteria to achieve the half confluent time (K/2) of EcM-WT and EcM-GDH in LB broth medium. K represents the maximum population. Data are expressed as means ± s.d. (n = 3, biological replicates).


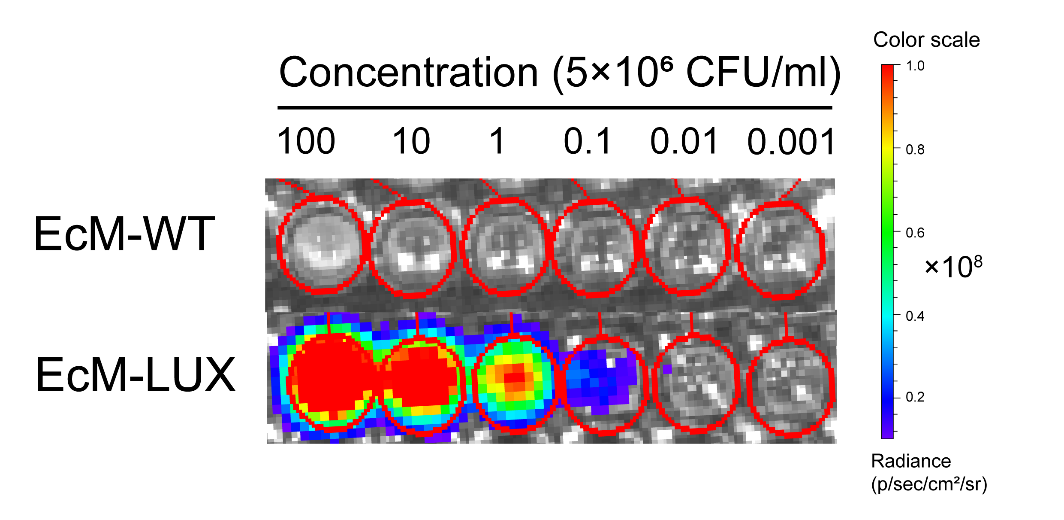


**Figure S10.** Luminescence detection of EcN (Lux) at varying concentrations.





**Figure S11**. The luminescence ratios of tumor/liver from the collected major organs and tumor tissue at 2 d, 4 d, and 8 d post-administration.





**Figure S12**. Tumor volume of mice from varied treatment groups during therapeutic evaluation.

**Table S1.** Amino acid sequences of the proteins involved in this study.

| **Protein Name** | **Protein Sequence** |
| --- | --- |
| GDH | MYPDLKGKVVAITGAASGLGKAMAIRFGKEQAKVVINYYSNKQDPNEVKEEVIKAGGEAVVVQGDVTKEEDVKNIVQTAIKEFGTLDIMINNAGLENPVPSHEMPLKDWDKVIGTNLTGAFLGSREAIKYFVENDIKGNVINMSSVHEVIPWPLFVHYAASKGGMKLMTRTLALEYAPKGIRVNNIGPGAINTTINKEKFADPEQRADVESMIPMGYIGEPEEIAAVAAWLASKEASYVTGITLFADGGMTLYPSFQAGRG |
| LUX-C | MTKKISFIINGQVEIFPESDDLVQSINFGDNSVYLPILNDSHVKNIIDCNGNNELRLHNIVNFLYTVGQRWKNEEYSRRRTYIRDLKKYMGYSEEMAKLEANWISMILCSKGGLYDVVENELGSRHIMDEWLPQDESYVRAFPKGKSVHLLAGNVPLSGIMSILRAILTKNQCIIKTSSTDPFTANALALSFIDVDPNHPITRSLSVIYWPHQGDTSLAKEIMRHADVIVAWGGPDAINWAVEHAPSYADVIKFGSKKSLCIIDNPVDLTSAATGAAHDVCFYDQRACFSAQNIYYMGNHYEEFKLALIEKLNLYAHILPNAKKDFDEKAAYSLVQKESLFAGLKVEVDIHQRWMIIESNAGVEFNQPLGRCVYLHHVDNIEQILPYVQKNKTQTISIFPWESSFKYRDALALKGAERIVEAGMNNIFRVGGSHDGMRPLQRLVTYISHERPSNYTAKDVAVEIEQTRFLEEDKFLVFVP* |
| LUX-D | MENESKYKTIDHVICVEGNKKIHVWETLPEENSPKRKNAIIIASGFARRMDHFAGLAEYLSRNGFHVIRYDSLHHVGLSSGTIDEFTMSIGKQSLLAVVDWLTTRKINNFGMLASSLSARIAYASLSEINASFLITAVGVVNLRYSLERALGFDYLSLPINELPDNLDFEGHKLGAEVFARDCLDFGWEDLASTINNMMYLDIPFIAFTANNDNWVKQDEVITLLSNIRSNRCKIYSLLGSSHDLSENLVVLRNFYQSVTKAAIAMDNDHLDIDVDITEPSFEHLTIATVNERRMRIEIENQAISLS* |
| LUX-A | MKFGNFLLTYQPPQFSQTEVMKRLVKLGRISEECGFDTVWLLEHHFTEFGLLGNPYVAAAYLLGATKKLNVGTAAIVLPTAHPVRQLEDVNLLDQMSKGRFRFGICRGLYNKDFRVFGTDMNNSRALAECWYGLIKNGMTEGYMEADNEHIKFHKVKVNPAAYSRGGAPVYVVAESASTTEWAAQFGLPMILSWIINTNEKKAQLELYNEVAQEYGHDIHNIDHCLSYITSVDHDSIKAKEICRKFLGHWYDSYVNATTIFDDSDQTRGYDFNKGQWRDFVLKGHKDTNRRIDYSYEINPVGTPQECIDIIQKDIDATGISNICCGFEANGTVDEIIASMKLFQSDVMPFLKEKQRSLLY* |
| LUX-B | MKFGLFFLNFINSTTVQEQSIVRMQEITEYVDKLNFEQILVYENHFSDNGVVGAPLTVSGFLLGLTEKIKIGSLNHIITTHHPVAIAEEACLLDQLSEGRFILGFSDCEKKDEMHFFNRPVEYQQQLFEECYEIINDALTTGYCNPDNDFYSFPKISVNPHAYTPGGPRKYVTATSHHIVEWAAKKGIPLIFKWDDSNDVRYEYAERYKAVADKYDVDLSEIDHQLMILVNYNEDSNKAKQETRAFISDYVLEMHPNENFENKLEEIIAENAVGNYTECITAAKLAIEKCGAKSVLLSFEPMNDLMSQKNVINIVDDNIKKYHMEYT* |
| LUX-E | MTSYVDKQEITASSEIDDLIFSSDPLVWSYDEQEKIRKKLVLDAFRNHYKHCREYRHYCQAHKVDDNITEIDDIPVFPTSVFKFTRLLTSQENEIESWFTSSGTNGLKSQVARDRLSIERLLGSVSYGMKYVGSWFDHQIELVNLGPDRFNAHNIWFKYVMSLVELLYPTTFTVTEERIDFVKTLNSLERIKNQGKDLCLIGSPYFIYLLCHYMKDKKISFSGDKSLYIITGGGWKSYEKESLKRDDFNHLLFDTFNLSDISQIRDIFNQVELNTCFFEDEMQRKHVPPWVYARALDPETLKPVPDGTPGLMSYMDASATSYPAFIVTDDVGIISREYGKYPGVLVEILRRVNTRTQKGCALSLTEAFDS* |

**Table S2.** Primer information for important genetic parts.

| **Genetic part name** | **DNA Sequence (5’-3’)** |
| --- | --- |
| GDH Forward | AAGCGGAATATATCCCTAGGATGTATCCGGATTTAAAAGGAAAAGTCG |
| GDH Reverse | GACAAATCCGCCGCCCTAGATTAGTGGTGGTGGTGGTGGTGACCGCGGCCTGCCTGGAATGAA |
| LUX Forward | ATGACTAAAAAAATTTCATTCATT |
| LUX Reverse | TCAACTATCAAACGCTTCGGTTAA |
| Vector Forward | ACATAAGCGTGCGATACAATACCCTGATTCTGTGGATAACCGTAGTCGACATAAGCGTGCGATACAATACCCTGATTCTGTGGATAACCGTAGTCG |
| Vector Reverse | GAATCAGGGTATTGTATCGCACGCTTATGTATTACTTAACAT |
